# Risk of Opioid Overdose Associated With Concomitant Use of Oxycodone and Selective Serotonin Reuptake Inhibitors

**DOI:** 10.1001/jamanetworkopen.2022.0194

**Published:** 2022-02-24

**Authors:** Ismaeel Yunusa, Joshua J. Gagne, Kazuki Yoshida, Katsiaryna Bykov

**Affiliations:** 1 Division of Pharmacoepidemiology and Pharmacoeconomics, Department of Medicine, Brigham and Women’s Hospital, Harvard Medical School, Boston, Massachusetts; 2 Center for Outcomes Research and Evaluation, Department of Clinical Pharmacy and Outcomes Sciences, University of South Carolina College of Pharmacy, Columbia; 3 Department of Epidemiology, Harvard T.H. Chan School of Public Health, Boston, Massachusetts; 4 Division of Rheumatology, Inflammation, and Immunity, Brigham and Women’s Hospital, Harvard Medical School, Boston, Massachusetts

## Abstract

**Importance:**

Some selective serotonin reuptake inhibitors (SSRIs) inhibit the enzymes responsible for the metabolism of oxycodone, a potent prescription opioid. The clinical consequences of this interaction on the risk of opioid overdose have not been elucidated.

**Objective:**

To compare opioid overdose rates in patients initiating oxycodone while taking SSRIs that are potent inhibitors of the cytochrome-P450 2D6 enzyme (CYP2D6) vs SSRIs that are not.

**Design, Setting, and Participants:**

This cohort study included adults who initiated oxycodone while receiving SSRI therapy between 2000 and 2020 whose data were included in 3 US health insurance databases.

**Exposures:**

Use of SSRIs that strongly inhibit CYP2D6 enzyme (fluoxetine or paroxetine) vs use of other SSRIs at the time of oxycodone initiation.

**Main Outcomes and Measures:**

Opioid overdose hospitalization or emergency department visit. Outcomes were assessed within 365 days of oxycodone initiation; in primary analyses, patients were followed up until the discontinuation of either oxycodone or their index SSRI group. Propensity score matching weights were used to adjust for confounding. Crude and weighted (adjusted) incidence rates and hazard ratios were estimated using Cox regression models, separately within each database and overall, stratifying on database.

**Results:**

A total of 2 037 490 initiated oxycodone while taking SSRIs (1 475 114 [72.4%] women; mean [SD] age, 50.1 [15.3] years). Most (1 418 712 [69.6%]) were receiving other SSRIs at the time of oxycodone initiation. In the primary analysis, we observed 1035 overdose events (0.05% of the study cohort). The adjusted incidence rate of opioid overdose in those using inhibiting SSRIs at the time of oxycodone initiation (9.47 per 1000 person-years) was higher than in those using other SSRIs (7.66 per 1000 person-years), indicating a greater risk of overdose among patients using CYP2D6-inhibiting SSRIs (adjusted hazard ratio, 1.23; 95% CI, 1.06-1.31). Results were consistent across multiple subgroup and sensitivity analyses.

**Conclusions and Relevance:**

In this cohort study of US adults, initiating oxycodone in patients treated with paroxetine or fluoxetine was associated with a small increased risk of opioid overdose.

## Key Points

**Question:**

Is concomitant use of oxycodone with selective serotonin reuptake inhibitors (SSRIs) that are potent inhibitors of oxycodone metabolism via the cytochrome-P450 2D6 (CYP2D6) enzyme (fluoxetine and paroxetine) associated with opioid overdose?

**Findings:**

In this cohort study of more than 2 million US adults, use of SSRIs that are potent inhibitors of oxycodone metabolism at the time of oxycodone therapy initiation was associated with a small but significantly higher risk of opioid overdose compared with the use of other, noninhibiting SSRIs.

**Meaning:**

These findings suggest that concomitant use of oxycodone with potent CYP2D6-inhibiting SSRIs may increase the risk of opioid overdose; other SSRIs should be considered for patients undergoing SSRI and oxycodone therapy.

## Teaser

This cohort study compares opioid overdose rates in patients initiating oxycodone while taking selective serotonin reuptake inhibitors (SSRIs) that are potent inhibitors of the cytochrome-P450 2D6 enzyme (CYP2D6) vs SSRIs that are not.

## Introduction

Opioid-related overdose deaths continue to impose an extensive and unabated public health burden in the United States.^1^ Between 1999 and 2019, more than half of all drug overdose deaths in the United States were attributed to opioids, with a substantial number involving prescription opioids.^2,3,4^ In 2019, nearly a third of all opioid overdose deaths occurred in people taking prescription opioids.^5,6^ Among the multiple risk factors associated with opioid overdose, a 2018 Surgeon General advisory warned that, even when opioid medications are used as prescribed, patients may be at higher risk of accidental overdose when using medications that can interact with opioids.^7^ The advisory specifically highlighted sedatives, such as benzodiazepines; however, opioids have the potential to interact, either pharmacokinetically or pharmacodynamically, with many other medications.^7,8,9,10^ For the most part, the clinical impact of pharmacological drug-drug interactions on opioid-related adverse events, including overdose, is unknown.

Selective serotonin reuptake inhibitors (SSRIs) are commonly coprescribed with opioids.^11,12^ Many conditions that cause pain, such as neuropathy, rheumatoid arthritis, and fibromyalgia, are also associated with depression.^13^ Studies also suggest that individuals with depression are more likely to develop new chronic pain that is subsequently treated with an opioid.^13,14,15,16,17^ SSRIs are the most commonly used class of antidepressants and one of the most commonly used drug classes overall in the United States.^18^ Some SSRIs, however, are potent inhibitors of liver enzymes responsible for metabolism of many medications, including oxycodone, a potent opioid. Oxycodone is metabolized by the cytochrome P450 (CYP) enzyme system, and 2 SSRIs, fluoxetine and paroxetine, are potent inhibitors of CYP2D6 enzymes.^19,20,21,22,23^ Oxycodone is one of the most commonly used opioids implicated in overdose deaths.^10^ Increased oxycodone plasma concentrations resulting from the interaction with fluoxetine or paroxetine could potentially lead to accidental overdose.^24,25,26^

Although pharmacokinetic studies have found increased plasma concentrations of oxycodone in the presence of CYP2D6 or CYP3A4 inhibition, no population-based study has examined whether concomitant use of oxycodone and CYP-inhibiting SSRIs is associated with opioid overdose.^27,28,29^ In this study, we investigated the comparative risk of opioid overdose in a large cohort of US patients who initiated oxycodone while taking SSRIs, comparing oxycodone initiation in the presence of SSRIs known to be potent or strong CYP2D6 inhibitors (fluoxetine and paroxetine) to oxycodone initiation in the presence of other SSRIs.

## Methods

We conducted a cohort study following the International Society for Pharmacoepidemiology Guidelines for Good Pharmacoepidemiology Practice.^30^ The article complies with the Reporting of Studies Conducted Using Observational Routinely Collected Health Data Statement for Pharmacoepidemiology (RECORD-PE) and the Strengthening the Reporting of Observational Studies in Epidemiology (STROBE) reporting guideline for cohort studies.^31,32^ The study was approved by the Brigham and Women’s Hospital’s Institutional Review Board, including a waiver for informed consent owing to the use of deidentified data.

### Data Sources and Settings

We used data from 3 large US health care claims databases covering individuals who received health insurance from commercial and public payers from 2000 to 2020. We specifically assembled deidentified data from Optum Clinformatics Data Mart (2004-2020), IBM Truven MarketScan (2003-2018), and Medicaid Analytic eXtract (MAX; 2000-2014). The deidentified Optum and MarketScan databases contain longitudinal medical and pharmacy claims for commercially insured and nationally representative US population. MAX includes medical and pharmacy claims for persons eligible for Medicaid in 49 US states and the District of Columbia.

### Study Design and Population

**Figure 1.**
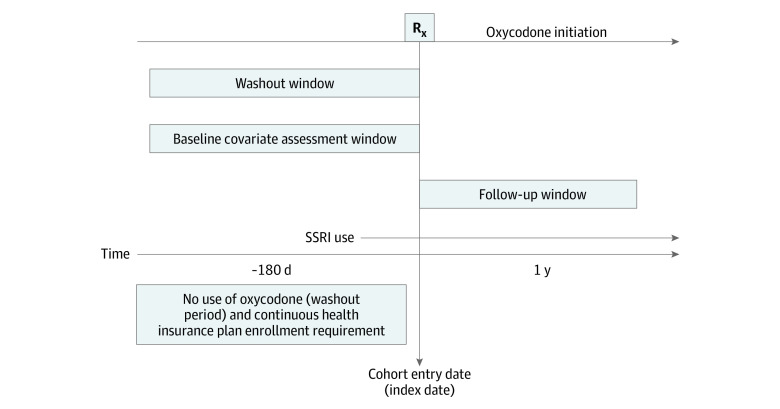
Visual Depiction of the Study Design Individuals taking selective serotonin reuptake inhibitors (SSRIs) with no oxycodone prescription in 180 days before their first oxycodone prescription were followed up from the day of oxycodone initiation. Patients were censored at the end of insurance enrollment, end of the database-specific study period, when switching to the other SSRI exposure group, when discontinuing either oxycodone or index SSRI exposure (defined as the end of days’ supply with no subsequent refilling within 14 days), or end of 1-year follow-up, whichever comes first. Rx indicates oxycodone prescription.

**Figure 2.**
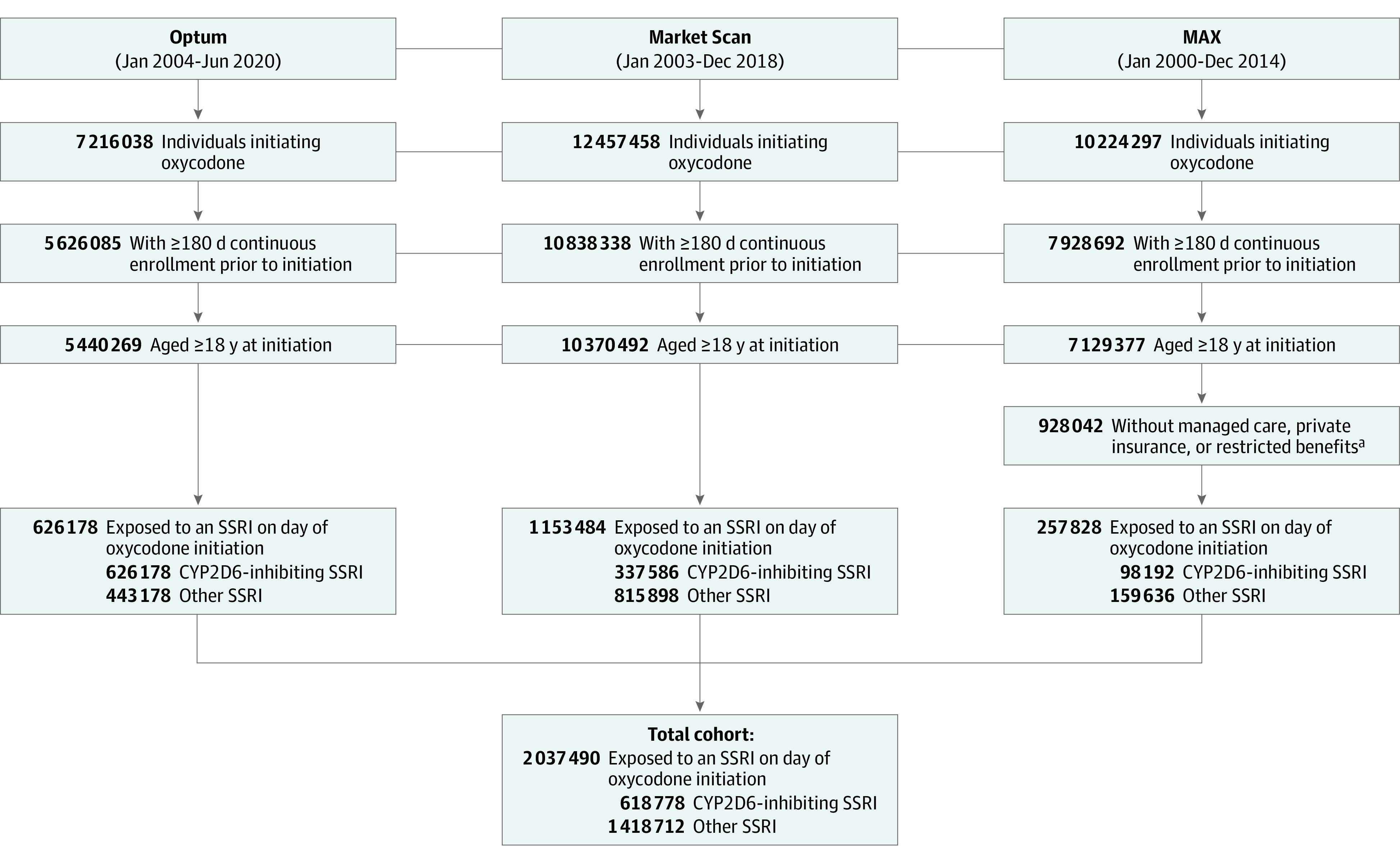
Cohort Selection Process CYP2D6 indicates cytochrome-P450 2D6; MAX, Medicaid Analytic eXtract; and SSRI, selective serotonin reuptake inhibitor. ^a^Medicaid patients with incomplete capture of claims, which encompasses patients with managed care plans, restricted benefits, or benefits administered through a private plan, were excluded.

Figure 1 presents a visual depiction of the study design, and Figure 2 depicts the study cohort selection process. The study population comprised adults aged 18 years or older who initiated oxycodone therapy while receiving SSRIs between 2000 and 2020. We defined initiation of oxycodone by requiring that patients have at least 180 days of oxycodone-free continuous enrollment in the database before their first prescription for oxycodone. We defined an active SSRI prescription as a dispensation before or on the date of oxycodone initiation with days’ supply overlapping the date of oxycodone initiation. We excluded Medicaid patients with incomplete capture of claims, such as those in managed care plans, restricted benefits, or benefits administered through a private plan.

### Exposure

We assessed medication exposure based on pharmacy-recorded dispensed fills, which included the date of dispensing, quantity dispensed, and days’ supply. Patients were divided into 2 exposure groups based on SSRI exposure on the date of oxycodone initiation. CYP2D6-inhibiting SSRIs included fluoxetine and paroxetine, SSRIs known to be potent inhibitors of CYP2D6.^19,20,21,22^ Other SSRIs included citalopram, escitalopram, fluvoxamine, and sertraline.

### Outcome and Follow-up

The outcome was hospitalization or emergency department visit for opioid overdose. We identified outcomes based on *International Classification of Diseases, Ninth Revision *(*ICD-9*) and *International Statistical Classification of Diseases and Related Health Problems, Tenth Revision *(*ICD-10*) codes for poisoning by opiates and related narcotics (eTable 1 in the Supplement). *ICD-9* codes used to define overdose, together with the code for poisoning by heroin, have been found to have a specificity of 99.9%.^33^

In the primary analysis, we followed up patients from the day of oxycodone initiation while receiving concomitant SSRI therapy until outcome occurrence, the end of insurance enrollment, end of data availability in the specific database, switching to the other SSRI exposure group, discontinuation of either oxycodone or index SSRI exposure (defined as the end of days’ supply with no subsequent refilling within 14 days), or end of 1 year of follow-up, whichever came first.

### Covariates

In total, we assessed 115 covariates that we believed to be risk factors, or proxies for risk factors, for opioid overdose. We assessed demographic characteristics (age, sex, year, and region in the United States) on the date of oxycodone initiation (index date). Comorbidities, prior medications, and health care utilization measures were assessed during the baseline period, defined as 180 days before the date of oxycodone initiation. Comorbidities included pain conditions, psychiatric disorders, depression, neurological disorders, cancer, diabetes, liver disease, and kidney dysfunction as well as opioid abuse or dependence, intentional self-harm, and any drug use disorder or drug dependence. We assessed prior use of antidepressants other than SSRIs, benzodiazepines, muscle relaxants, antipsychotics, anticonvulsants, dementia drugs, triptans, and nonsteroidal anti-inflammatory drugs. Measures of health care utilization included number of physician visits and number of hospitalizations as well as hospitalization in the 30 days prior to oxycodone initiation and the number of unique drugs dispensed during the baseline period. We estimated the amount of prior nonoxycodone opioid exposure using morphine milligram equivalents (MMEs) as well as total oxycodone amount and nonoxycodone opioid amount dispensed on the index date (also in MMEs). Finally, we assessed exposure to other drugs that are known to affect CYP2D6 and CYP3A4 enzymes (eTable 2 in the Supplement) on the index date and estimated chronic disease burden with the combined comorbidity score (a combination of conditions in the Charlson and Elixhauser measures that better estimates patient mortality).^34^

### Statistical Analysis

To control for confounding due to nonrandom allocation of patients to treatment groups, we used propensity score (PS) matching weights (MW). The MW approach represents an extension of the inverse probability of treatment weighting (IPTW) that mimics PS matching and standardizes the distribution of covariates to those of patients in empirical equipoise.^35^ Weights are bound between 0 and 1, reducing the consequences of extreme weights in comparison to IPTW.^36^ We used logistic regression models to estimate patients’ probabilities of exposure to inhibiting SSRIs vs other SSRIs on the index date as a function of all pretreatment covariates.^37,38^ We fit a separate PS model within each database. Following PS estimation, we calculated MWs as the smaller of the estimated probabilities of receiving or not receiving inhibiting SSRIs divided by the estimated probability of being assigned to the exposure group a patient was actually in.^36^ We assessed covariate balance between treatment groups before and after weighting, using standardized differences.^39^ A standardized difference greater than 0.1 was considered an imbalance.^39^

We report crude (unweighted) and adjusted (weighted) numbers of events and incidence rates (IR) per 1000 person-years of follow-up for each treatment group. We used Cox proportional hazards models^40^ to estimate the crude and weighted (ie, adjusted) hazard ratios (HRs) and 95% CIs, comparing potent CYP2D6-inhibiting SSRIs with other SSRIs. We conducted these analyses within each database and in all data pooled from the 3 databases using a Cox regression model, stratified by database. Statistical significance was established by the 95% CIs: if the 95% CI did not include 1, it was considered significant; if it did include 1, it was considered not significant. All analyses were performed using R version 4.1.0. (R Project for Statistical Computing).

To examine the robustness of our findings and account for potential differential informative censoring, we conducted a sensitivity analysis using an intention-to-treat (ITT) approach.^41,42^ In this analysis, we followed up patients for a maximum of 60 days from oxycodone initiation, regardless of changes in therapy. Patients were followed from the day of oxycodone initiation until the first of outcome occurrence, the end of insurance enrollment, the end of data availability in the specific database, or end of 60 days follow-up.

Additionally, to assess potential effect modification, we conducted subgroup analyses, stratified by pertinent patient characteristics, such as age (<65 years vs ≥65), sex, total MME dispensed on the index date (categorized as less than the median value vs the median or greater), and prior opioid use (defined as any nonoxycodone opioid dispensing during the baseline period). In these analyses, we reestimated database-specific PSs and MWs within each specific subgroup of interest and pooled the data from the 3 databases, as described previously. Unless otherwise noted, sensitivity and subgroup analyses of the outcome model were conducted with the same statistical approaches used in the primary analysis.

## Results

### Study Population

We identified 2 037 490 individuals who were receiving SSRI therapy at the time of oxycodone initiation; 618 778 (30.4%) were receiving inhibiting SSRIs (paroxetine, 243 787 [12.0%]; fluoxetine, 374 991 [18.4%]) and 1 418 712 (69.6%) were receiving other SSRIs (citalopram, 407 501 [20.0%]; escitalopram, 449 549 [22.1%]; fluvoxamine, 9142 [0.4%]; sertraline, 552 520 [27.1%]). Baseline characteristics were well balanced between the 2 treatment groups before and after PS weighting (Table 1). eTable 3 in the Supplement presents the complete list of baseline characteristics with standardized differences, and eTables 4 to 6 in the Supplement present database-specific characteristics. The mean (SD) age of patients was 50.1 (15.3) years, and 1 475 114 (72.4%) were women.

**Table 1.** Selected Patient Baseline Characteristics Before and After Propensity Score Matching Weighting

Patient characteristic	Unweighted			Weighted		
	Patients by SSRI group, No. (%)		Standardized difference^a^	Patients by SSRI group, No. (%)		Standardized difference^a^
	CYP2D6-inhibiting SSRI (n = 618 778)	Other SSRI (n = 1 418 712)		CYP2D6-inhibiting SSRI (n = 614 385)	Other SSRI (n = 614 336)	
Demographic characteristics						
Age, mean (SD), y	50.09 (14.66)	50.03 (15.54)	0.004	50.13 (14.68)	50.08 (14.75)	0.003
Women	457 181 (73.9)	1 017 933 (71.8)	0.049	453 481 (73.8)	453 318 (73.8)	<0.001
Men	161 396 (26.1)	400 463 (28.2)		160 714 (26.2)	160 829 (26.2)	
Comorbidities						
Combined comorbidity score, mean (SD)	0.85 (1.82)	0.97 (2.01)	0.063	0.85 (1.82)	0.85 (1.83)	0.001
Alcohol use disorder or dependence	7534 (1.2)	17 995 (1.3)	0.005	7454 (1.2)	7478 (1.2)	<0.001
Anxiety	145 133 (23.5)	368 942 (26.0)	0.059	144 172 (23.5)	144 379 (23.5)	0.001
Back and neck pain	213 302 (34.5)	480 401 (33.9)	0.013	211 468 (34.4)	211 825 (34.5)	0.001
Bipolar disorder	29 533 (4.8)	55 819 (3.9)	0.041	29 156 (4.7)	29 530 (4.8)	0.001
Back pain						
Without radiculopathy	193 753 (31.3)	433 119 (30.5)	0.017	191 986 (31.2)	192 272 (31.3)	0.001
With radiculopathy	56 462 (9.1)	127 185 (9.0)	0.006	56 116 (9.1)	56 221 (9.1)	0.001
Bone fracture	14 378 (2.3)	41 851 (2.9)	0.039	14 377 (2.3)	14 385 (2.3)	<0.001
Cancer	50 887 (8.2)	128 400 (9.1)	0.029	50 656 (8.2)	50 570 (8.2)	<0.001
COPD, asthma, or oxygen use	92 373 (14.9)	207 671 (14.6)	0.008	91 458 (14.9)	91 499 (14.9)	<0.001
Dementia	11 966 (1.9)	42 181 (3.0)	0.067	11 954 (1.9)	11 957 (1.9)	<0.001
Dental pain	12 352 (2.0)	23 015 (1.6)	0.028	12 006 (2.0)	12 052 (2.0)	0.001
Depression	178 850 (28.9)	434 992 (30.7)	0.038	177 706 (28.9)	178 363 (29.0)	0.002
Diabetes	94 277 (15.2)	215 056 (15.2)	0.002	93 587 (15.2)	93 498 (15.2)	<0.001
Diabetic neuropathy	14 476 (2.3)	35 465 (2.5)	0.01	14 399 (2.3)	14 413 (2.3)	<0.001
Epilepsy or convulsions	15 649 (2.5)	35 218 (2.5)	0.003	15 404 (2.5)	15 459 (2.5)	0.001
Fibromyalgia	42 235 (6.8)	87 819 (6.2)	0.026	41 791 (6.8)	42 011 (6.8)	0.001
Headache	87 642 (14.2)	195 453 (13.8)	0.011	86 759 (14.1)	87 054 (14.2)	0.001
Intentional self-harm	1684 (0.3)	4036 (0.3)	0.002	1676 (0.3)	1680 (0.3)	<0.001
Liver disease	37 041 (6.0)	89 621 (6.3)	0.014	36 761 (6.0)	36 812 (6.0)	0.001
Musculoskeletal injury	76 285 (12.3)	175 443 (12.4)	0.001	75 766 (12.3)	75 906 (12.4)	0.001
Opioid use disorder or dependence	7582 (1.2)	14 835 (1.0)	0.017	7445 (1.2)	7466 (1.2)	<0.001
Osteoarthritis	107 848 (17.4)	248 164 (17.5)	0.002	107 272 (17.5)	107 210 (17.5)	<0.001
Other arthritis, arthropathies, and musculoskeletal pain	283 302 (45.8)	659 259 (46.5)	0.014	281 350 (45.8)	281 579 (45.8)	0.001
Other neuropathic pain	109 214 (17.6)	249 634 (17.6)	0.001	108 505 (17.7)	108 727 (2.3)	0.001
Other drug use disorder or dependence	14 612 (2.4)	28 573 (2.0)	0.024	14 345 (2.3)	14 410 (2.3)	0.001
Postherpetic neuralgia	830 (0.1)	2162 (0.2)	0.005	823 (0.1)	816 (0.1)	<0.001
Previous overdose	891 (0.1)	1699 (0.1)	0.007	870 (0.1)	868 (0.1)	<0.001
Psychosis	14 132 (2.3)	29 951 (2.1)	0.012	13 856 (2.3)	13 931 (2.3)	0.001
Kidney dysfunction	38 050 (6.1)	99 132 (7.0)	0.034	37 934 (6.2)	37 890 (6.2)	<0.001
Rheumatoid arthritis	12 434 (2.0)	27 110 (1.9)	0.007	12 319 (2.0)	12 342 (2.0)	<0.001
Tobacco use	71 124 (11.5)	167 210 (11.8)	0.009	70 807 (11.5)	70 901 (11.5)	0.001
Urinary calculus	31 176 (5.0)	75 031 (5.3)	0.011	31 026 (5.1)	31 100 (5.1)	0.001
Opioid-related medication use, mean (SD)						
Total oxycodone dispensed on index date, MME	423.94 (554.70)	413.14 (539.64)	0.020	429.77 (595.47)	430.12 (598.12)	0.001
Total nonoxycodone dispensed on index date, MME	25.64 (136.46)	23.89 (130.97)	0.013	28.52 (160.72)	28.56 (160.60)	<0.001
Total dispensed 60 d prior to index date, MME	559.93 (1464.62)	484.04 (1364.58)	0.054	583.96 (1644.74)	584.43 (1655.84)	<0.001
Total dispensed 180 d prior to index date, MME	1571.82 (4243.76)	1357.44 (3947.98)	0.052	1633.31 (4723.77)	1635.05 (4759.39)	<0.001
Other prior medications						
Other antidepressants	162 188 (26.2)	344 128 (24.3)	0.045	160 391 (26.1)	161 212 (26.2)	0.003
Benzodiazepines	229 768 (37.1)	508 449 (35.8)	0.027	227 523 (37.0)	228 132 (37.1)	0.002
Muscle relaxants	140 775 (22.8)	299 501 (21.1)	0.040	139 431 (22.7)	139 735 (22.7)	0.001
Other sedatives or hypnotics	83 542 (13.5)	198 287 (14.0)	0.014	83 046 (13.5)	83 354 (13.6)	0.001
NSAID	238 226 (38.5)	522 889 (36.9)	0.034	235 713 (38.4)	235 789 (38.4)	<0.001
Lithium	5580 (0.9)	9210 (0.6)	0.029	5436 (0.9)	5516 (0.9)	0.001
Antipsychotics						
Atypical	54 791 (8.9)	105 438 (7.4)	0.052	54 107 (8.8)	54 632 (8.9)	0.003
Typical	4717 (0.8)	8682 (0.6)	0.018	4549 (0.7)	4577 (0.7)	0.001
Barbiturates	3311 (0.5)	5700 (0.4)	0.020	3233 (0.5)	3235 (0.5)	<0.001
Agents for dementia	5786 (0.9)	22 194 (1.6)	0.057	5782 (0.9)	5783 (0.9)	<0.001
Anticonvulsants	63 887 (10.3)	136 013 (9.6)	0.025	63 305 (10.3)	63 908 (10.4)	0.003
Gabapentinoids	81 383 (13.2)	183 276 (12.9)	0.007	80 791 (13.1)	81 117 (13.2)	0.002
Triptans	29 940 (4.8)	62 457 (4.4)	0.021	29 667 (4.8)	29 850 (4.9)	0.001
CYP3A4, on index date						
Inhibitors	52 695 (8.5)	119 390 (8.4)	0.004	52 234 (8.5)	52 192 (8.5)	<0.001
Inducers	29 604 (4.8)	62 288 (4.4)	0.019	29 329 (4.8)	29 583 (4.8)	0.002
CYP2D6, on index date						
Inhibitors	6769 (1.1)	16 020 (1.1)	0.003	6707 (1.1)	6674 (1.1)	<0.001
Inducers	9539 (1.5)	18 944 (1.3)	0.017	9355 (1.5)	9334 (1.5)	<0.001
Health care utilization, mean (SD)						
Distinct drugs, No.	9.44 (5.86)	9.15 (5.78)	0.049	9.41 (5.85)	9.43 (5.86)	0.002
Physician visits, No.	5.33 (4.31)	5.44 (4.34)	0.026	5.33 (4.31)	5.34 (4.27)	0.003
Hospitalizations, No.	0.34 (0.77)	0.37 (0.85)	0.033	0.34 (0.77)	0.34 (0.76)	0.001
Hospitalization in 30 d before index, No. (%)	118 630 (19.2)	285 570 (20.1)	0.024	117 942 (19.2)	117 540 (19.1)	0.002

Abbreviations: COPD, chronic obstructive pulmonary disease; CYP, cytochrome P450; MME, morphine milligram equivalent; NSAID, nonsteroidal anti-inflammatory drugs; SSRI, selective serotonin reuptake inhibitor.

^a^ Standardized differences greater than 0.1 indicate lack of balance between exposure groups.

### Opioid Overdose

The mean duration of concomitant exposure to both oxycodone and an SSRI was 23 days, regardless of exposuire group, and did not differ substantially between the 2 exposure groups (eTable 7 in the Supplement). Most patients (1 507 730 [77.1%]) were censored because they discontinued oxycodone; 482 507 patients (27.2%) were censored because of SSRIs discontinuation (eTable 8 in the Supplement). In the primary analysis, we observed 1035 overdose events (0.05% of the study cohort). We observed 654 crude overdose events during 90 125 person-years of follow-up in the group exposed to other SSRIs and 381 events during 40 053 person-years of follow-up in the group exposed to inhibiting SSRIs, yielding an unadjusted HR of 1.20 (95% CI, 1.06-1.36) for inhibiting SSRIs vs other SSRIs (Table 2). The adjusted incidence rate of opioid overdose for CYP2D6-inhibiting SSRIs (9.47 per 1000 person-years) was higher compared with other SSRIs (7.66 per 1000 person-years). The pooled adjusted analysis revealed that coadministration of oxycodone with CYP2D6-inhibiting SSRIs was associated with a significantly greater hazard of opioid overdose compared with coadministration with other SSRIs (adjusted HR, 1.23; 95% CI, 1.06-1.31). The rate of opioid overdose in Optum and MarketScan databases were comparable, while that in Medicaid population was higher (eTable 9 in the Supplement). All database-specific estimates suggested concordant direction of the association.

**Table 2.** Risk of Opioid Overdose Associated With Concomitant Use of Oxycodone and CYP2D6-Inhibiting SSRIs vs Oxycodone and Other SSRIs

Exposure	No.		Follow-up, person-years	Incidence rate, per 1000 person-years		HR (95% CI)	
	Patients	Events		Unadjusted	Adjusted	Unadjusted	Adjusted
**Primary analysis^a^**							
Other SSRI	1 418 712	654	90 125	7.26	7.66	1 [Reference]	1 [Reference]
CPY2D6-inhibiting SSRI	618 778	381	40 053	9.51	9.47	1.20 (1.06-1.36)	1.23 (1.06-1.31)
**Intention-to-treat analysis**							
Other SSRI	1 418 712	996	224 766	4.43	4.82	1 [Reference]	1 [Reference]
CPY2D6-inhibiting SSRI	618 778	559	98 219	5.69	5.66	1.18 (1.06-1.31)	1.17 (1.05-1.30)

Abbreviations: CYP, cytochrome P450; HR, hazard ratio; SSRI, selective serotonin reuptake inhibitor.

^a^ Patients were censored on discontinuation of either oxycodone or index SSRI exposure group and on switch to the other treatment group.

### Sensitivity and Subgroup Analyses

**Figure 3.**
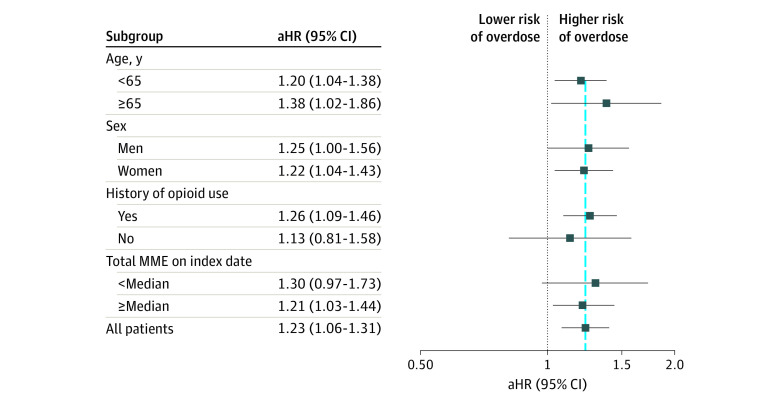
Association Between Concomitant Exposure to Selective Serotonin Reuptake Inhibitors and Oxycodone and Opioid Overdose Among Subgroups The vertical blue line shows the overall measure of association in all patients. aHR indicates adjusted hazard ratio; MME, morphine milligram equivalent.

In the 60-day ITT analysis in which patients were not censored on treatment changes, the overall pooled estimate was consistent with the primary analysis estimate (adjusted HR, 1.17; 95% CI, 1.05-1.30). Subgroup analyses showed consistent results. Among older adults (≥65 years), there was an adjusted HR of 1.38 (95% CI, 1.02-1.86) for CYP2D6-inhibiting SSRIs vs other SSRIs (Figure 3).

## Discussion

In this large cohort study of a representative sample of more than 2 million patients, starting oxycodone while receiving a CYP2D6-inhibiting SSRI (fluoxetine or paroxetine) vs other SSRIs was associated with a small but significant increase in the risk of opioid overdose. The overall occurrence of outcomes was low, with only 0.05% of patients experiencing an overdose that led to an emergency department visit or hospitalization while receiving concomitant oxycodone-SSRI therapy. Nevertheless, these findings add to our knowledge regarding factors that may contribute to opioid overdoses in adults taking multiple medications.

Prior studies examining the potential for pharmacokinetic drug interaction between CYP2D6-inhibiting SSRIs and oxycodone have produced mixed findings.^29,43^ Although some pharmacokinetic studies have found that inhibiting the CYP2D6 enzyme can increase oxycodone plasma concentration, others have not observed such an increase.^43,44,45^ To our knowledge, the current study is the first population-based, real-world study assessing the clinical consequences of this potential interaction. A study that examined outcomes of prescribing hydrocodone, another commonly used opioid analgesic, recommended that for patients taking CYP2D6-inhibiting SSRIs, prescribers select oxycodone or morphine as a more effective alternative, because the authors found that inhibiting CYP2D6 was associated with suboptimal pain control with hydrophone.^14^ However, the current study’s findings suggest that oxycodone may not be an optimal substitute in the presence of CYP2D6-inhibiting SSRIs.

Given that most studies suggest the central effects of oxycodone on both analgesia and respiratory depression are attributed more to parent oxycodone than to its metabolites,^46,47^ the higher overdose rate found in our study may have been caused, at least partially, by increased oxycodone plasma concentration from the inhibition of its metabolism by paroxetine or fluoxetine. In addition to strongly inhibiting CYP2D6, both paroxetine and fluoxetine mildly inhibit CYP3A4, which may further increase oxycodone concentration in blood.^20,23,48^ The narrow therapeutic index of oxycodone may have contributed to the increased risk of overdose.^49,50,51^ It has been demonstrated that concomitant use of potent inhibitors of CYP2D6 with substrates that have a narrow therapeutic index is more likely to be associated with clinically relevant adverse health outcomes than substrates with a wide therapeutic index.^52^

Of note, the magnitude of the observed potential interaction in our study is small. Our results suggest that the interaction does not appear to manifest in detrimental clinical outcomes for most patients. Moreover, the overall incidence of overdoses was very low. However, we may have not captured all overdose events that occurred in our cohort because of a highly specific outcome definition that required a hospitalization or emergency department visit. In addition, our study focused on overdoses only; it is possible that the interaction might lead to higher incidence of other, more common oxycodone adverse events, such as drowsiness, confusion, or constipation. While the use of oxycodone has generally decreased from 2014, it is still relatively high.^53^ Moreover, the rate of overdoses that involve oxycodone has remained relatively high.^54^ Given that most SSRIs have similar antidepressant effectiveness profiles, it is possible that unintentional opioid overdose events can be prevented by considering an SSRI that is not a potent inhibitor of CYP2D6.

Our findings of a potentially more pronounced increase in overdose risk in persons aged 65 years and older have important implications for routine clinical practice. Possible age-related decreases in clearance of oxycodone^55^ might increase the risk of overdose in this population. Also, older adults may be more susceptible to adverse effects of opioids and may be taking many other medications that further increase the risk of overdose from drug interactions. Further studies are needed to confirm that older adults indeed have a heightened risk of overdose or other adverse events because of oxycodone drug interactions.

### Limitations

This study’s findings should be interpreted with the following limitations in mind. First, administrative claims data do not provide information on clinical parameters that may affect outcomes, such as CYP2D6 genotype, which may act as an important effect modifier.^52,56^ We also did not have information on some important overdose risk factors, such as illicit drug use and socioeconomic status. It is worth noting, however, that unmeasured risk factors would only confound the association in our study if they were associated with the choice of an SSRI, once the measured variables are controlled for. Given that even before adjustment, the measured baseline characteristics were well balanced between the 2 exposure groups, it is unlikely that the choice of an SSRI was driven by characteristics that would represent a major risk factor for opioid overdose. Moreover, prior studies have shown that adjusting for numerous clinical and health care utilization variables that are available in administrative health care claims databases often balances important, but unmeasured, confounders by proxy.^57^ Nevertheless, as in any observational study, residual confounding cannot be excluded.

Misclassification of exposure is possible in claims-based analyses because pharmacy claim records provide accurate information about reimbursed drugs dispensed to patients but do not provide information on whether patients actually took the medications as prescribed. Claims-based outcome definitions are also subject to potential misclassification. While we used a highly specific outcome definition,^33^ which ensures an unbiased measure of relative risk, our definition may have missed events that did not result in contact with health care system, and thus, our incidence rates may be underestimated. *ICD*-based classification also precludes evaluation of opioid-specific overdoses. Thus, it is possible that some of the outcomes in our cohort were caused by opioids other than oxycodone, including illicit drugs. Moreover, the duration of follow-up was relatively short in our study, as most people only had short oxycodone therapy. Future studies could investigate how the risk changes with longer concomitant duration. The data use agreement with Optum prevents us from knowing the exact degree of potential overlap in the deidentified sample of patients between Optum and MarketScan databases. Furthermore, it should also be noted that because the absolute magnitude of the association observed in our study is statistically significant but small with a narrow confidence interval, we cannot completely rule out a chance finding. Future research may be needed to confirm our findings.

## Conclusions

In this cohort study of US adults, initiation of oxycodone while receiving paroxetine or fluoxetine therapy was associated with an increased risk of opioid overdose compared with oxycodone initiation while receiving other SSRIs. The absolute magnitude of the observed association in our study is small, and further research is needed to corroborate our findings.

## Notes

Supplement. **eTable 1. **Outcome Definitions **eTable 2.** List of Drugs That Inhibit or Induce Cytochrome P450 2D6 or 3A4 Enzymes Adjusted for in the Propensity Score Model **eTable 3. **Patient Baseline Characteristics Before and After Propensity Score Weighting for the Combined Database (Optum, MarketScan, and MAX) **eTable 4.** Patient Baseline Characteristics Before and After Propensity Score Weighting for MarketScan Database **eTable 5. **Patient Baseline Characteristics Before and After Propensity Score Weighting for MAX Database **eTable 6. **Patient Baseline Characteristics Before and After Propensity Score Weighting for Optum Database **eTable 7. **Follow-up Duration **eTable 8. **Reasons for Censoring by Treatment Group Based on Primary Analysis **eTable 9.** Results for Each Database
