# Supplementary material for: Risk of Opioid Overdose Associated With Concomitant Use of Oxycodone and Selective Serotonin Reuptake Inhibitors
Source: JAMA Netw Open. 2022 Feb 24;5(2):e220194. doi: 10.1001/jamanetworkopen.2022.0194 (PMC8874341; doi:10.1001/jamanetworkopen.2022.0194)
Supplement: Supplement. — eTable 1. Outcome Definitions eTable 2. List of Drugs That Inhibit or Induce Cytochrome P450 2D6 or 3A4 Enzymes Adjusted for in the Propensity Score Model eTable 3. Patient Baseline Characteristics Before and After Propensity Score Weighting for the Combined Database (Optum, MarketScan, and MAX) eTable 4. Patient Baseline Characteristics Before and After Propensity Score Weighting for MarketScan Database eTable 5. Patient Baseline Characteristics Before and After Propensity Score Weighting for MAX Database eTable 6. Patient Baseline Characteristics Before and After Propensity Score Weighting for Optum Database eTable 7. Follow-up Duration eTable 8. Reasons for Censoring by Treatment Group Based on Primary Analysis eTable 9. Results for Each Database [file jamanetwopen-e220194-s001.pdf]

Supplemental Online Content

Yunusa I, Gagne JJ, Yoshida K, Bykov K. Risk of opioid overdose associated with concomitant use of oxycodone and selective serotonin reuptake inhibitors. *JAMA Netw Open.* 2022;5(2):e220194. doi:10.1001/jamanetworkopen.2022.0194

eTable 1. Outcome Definitions

eTable 2. List of Drugs That Inhibit or Induce Cytochrome P450 2D6 or 3A4 Enzymes Adjusted for in the Propensity Score Model

eTable 3. Patient Baseline Characteristics Before and After Propensity Score Weighting for the Combined Database (Optum, MarketScan, and MAX)

eTable 4. Patient Baseline Characteristics Before and After Propensity Score Weighting for MarketScan Database

eTable 5. Patient Baseline Characteristics Before and After Propensity Score Weighting for MAX Database

eTable 6. Patient Baseline Characteristics Before and After Propensity Score Weighting for Optum Database

eTable 7. Follow-up Duration

eTable 8. Reasons for Censoring by Treatment Group Based on Primary Analysis

eTable 9. Results for Each Database

This supplemental material has been provided by the authors to give readers additional information about their work.

© 2022 Yunusa I et al. *JAMA Network Open.*

eTable 1. Outcome Definitions

**Diagnosis code**	**Notes**
**ICD10** (last digit * stands for any, which will be A or D or S)	
T400X1*	Accidental poisoning by opium
T400X2*	Poisoning by opium, intentional self-harm
T400X3*	Poisoning by opium, assault
T400X4*	Poisoning by adverse effect of and underdosing of opium
T402X1*	Accidental poisoning by other opioids
T402X2*	Poisoning by other opioids, intentional self-harm
T402X3*	Poisoning by other opioids, assault
T402X4*	Poisoning by other opioids, undetermined.
T403X1*	Accidental poisoning by methadone
T403X2*	Poisoning by methadone, intentional self-harm
T403X3*	Poisoning by methadone, assault
T403X4*	Poisoning by methadone, undetermined
T404X1*	Accidental poisoning by other synthetic narcotics
T404X2*	Poisoning by other synthetic narcotics, intentional self-harm
T404X3*	Poisoning by other synthetic narcotics, assault
T404X4*	Poisoning by other synthetic narcotics, undetermined
T40601*	Accidental poisoning by unspecified narcotics
T40602*	Poisoning by unspecified narcotics, intentional self-harm
T40603*	Poisoning by unspecified narcotics, assault
T40604*	Poisoning by unspecified narcotics, undetermined
T40691*	Accidental poisoning by other narcotics
T40692*	Poisoning by other narcotics, intentional self-harm
T40693*	Poisoning by other narcotics, assault
T40694*	Poisoning by other narcotics, undetermined
**ICD9**	
965.0x	Opiate Poisonings
E850.1	Accidental poisoning by methadone
E850.2	Accidental poisoning by other opiates and related narcotics

© 2022 Yunusa I et al. *JAMA Network Open.*

eTable 2. List of Drugs That Inhibit or Induce Cytochrome P450 2D6 or 3A4 Enzymes Adjusted for in the Propensity Score Model

CYP 3A4 Inhibitors	CYP 3A4 Inducers	CYP 2D6 Inhibitors	CYP 2D6 Inducers
Atazanavir	Etravirine	Amiodarone	Carbamazepine
Boceprevir	Nevirapine	Cimetidine	Phenobarbital
Darunavir	Nelfinavir	Propafenone	Phenytoin
Delavirdine	Aprobarbital	Clomipramine	Rifampin
Efavirenz	Amobarbital	Quinidine	Ritonavir
Fosamprenavir	Butabarbital	Desipramine	
Indinavir	Pentobarbital	Ritonavir	
Lopinavir	Secobarbital	Fluphenazine	
Nelfinavir	Bosentan	Thioridazine	
Ritonavir	Carbamazepine	Haloperidol	
Saquinavir	Fosphenytoin		
Telaprevir	Modafinil		
Itraconazole	Oxcarbazepine		
Ketoconazole	Phenobarbital		
Posaconazole	Phenytoin		
Voriconazole	Primidone		
Cannabidiol	Rifampin		
Imatinib	Rifabutin		
Isoniazid	Clobazam		
Quinidine	Ethosuximide		
Clarithromycin	Phenylbutazone		
Telithromycin	Tipranavir		
Amiodarone	Topiramate		
Amprenavir			
Aprepitant			
Cimetidine			
Cyclosporine			
Desipramine			
Diltiazem			
Doxycycline			
Dronedarone			
Erythromycin			
Fluconazole			
Haloperidol			
Lapatinib			
Lidocaine			
Miconazole			
Norfloxacin,			
Tetracycline			
Verapamil			

Abbreviation: CYP, cytochrome P450

© 2022 Yunusa I et al. *JAMA Network Open.*

eTable 3. Patient Baseline Characteristics Before and After Propensity Score Weighting for the Combined Database (Optum, MarketScan, and MAX)

Patient characteristic	SSRI Exposure groups[a]					
	Unweighted			Weighted		
	CPY2D6-inhibiting SSRI	Other SSRI	[b]Std. diff.	Inhibiting SSRI	Other SSRI	[b]Std. diff.
**Number of patients**	618 778	1 418 712		614 385	614 336	
**Demographics**						
Age, mean (SD), y	50.09 (14.66)	50.03 (15.54)	0.004	50.13 (14.68)	50.08 (14.75)	0.003
Age category, y			0.097			0.002
18-24	29 761 (4.8)	74 598 (5.3)		29 628 (4.8)	29 746 (4.8)	
25-34	68 530 (11.1)	177 469 (12.5)		67 947 (11.1)	67 947 (11.1)	
35-44	117 207 (18.9)	270 245 (19.0)		115 927 (18.9)	115 997 (18.9)	
45-54	158 285 (25.6)	335 066 (23.6)		156 720 (25.5)	156 978 (25.6)	
55-64	149 616 (24.2)	322 090 (22.7)		148 820 (24.2)	148 669 (24.2)	
65-74	63 469 (10.3)	142 309 (10.0)		63 431 (10.3)	63 257 (10.3)	
75 and above	31 910 (5.2)	96 935 (6.8)		31 909 (5.2)	31 740 (5.2)	
Sex			0.049			<0.001
Female	457 181 (73.9)	1 017 933 (71.8)		453 481 (73.8)	453 318 (73.8)	
Region			0.06			0.001
Midwest	135 601 (21.9)	318 410 (22.4)		134 792 (21.9)	134 895 (22.0)	
Northeast	102 861 (16.6)	249 800 (17.6)		102 129 (16.6)	102 152 (16.6)	
South	258 766 (41.8)	602 824 (42.5)		257 242 (41.9)	257 383 (41.9)	
West	119 074 (19.2)	241 304 (17.0)		117 748 (19.2)	117 430 (19.1)	
Unknown	2476 (0.4)	6374 (0.4)		2472 (0.4)	2474 (0.4)	
Calendar year of index date, n (%)			0.271			0.001
2000	5042 (0.8)	3726 (0.3)		3702 (0.6)	3703 (0.6)	
2001	10 068 (1.6)	8196 (0.6)		8090 (1.3)	8085 (1.3)	
2002	9766 (1.6)	9562 (0.7)		9010 (1.5)	9013 (1.5)	
2003	22 657 (3.7)	28 688 (2.0)		22 454 (3.7)	22 470 (3.7)	

© 2022 Yunusa I et al. *JAMA Network Open.*

2004	37 800 (6.1)	55 441 (3.9)		37 717 (6.1)	37 709 (6.1)	
2005	37 928 (6.1)	60 917 (4.3)		37 903 (6.2)	37 895 (6.2)	
2006	37 773 (6.1)	68 453 (4.8)		37 765 (6.1)	37 725 (6.1)	
2007	35 914 (5.8)	72 497 (5.1)		35 912 (5.8)	35 854 (5.8)	
2008	39 764 (6.4)	86 842 (6.1)		39 763 (6.5)	39 727 (6.5)	
2009	43 617 (7.0)	103 101 (7.3)		43 616 (7.1)	43 622 (7.1)	
2010	43 420 (7.0)	106 930 (7.5)		43 420 (7.1)	43 401 (7.1)	
2011	46 884 (7.6)	119 502 (8.4)		46 883 (7.6)	46 874 (7.6)	
2012	45 462 (7.3)	119 683 (8.4)		45 462 (7.4)	45 504 (7.4)	
2013	37 619 (6.1)	101 936 (7.2)		37 619 (6.1)	37 626 (6.1)	
2014	35 066 (5.7)	97 388 (6.9)		35 066 (5.7)	35 065 (5.7)	
2015	30 398 (4.9)	84 960 (6.0)		30 398 (4.9)	30 405 (4.9)	
2016	30 539 (4.9)	86 853 (6.1)		30 539 (5.0)	30 561 (5.0)	
2017	28 123 (4.5)	81 616 (5.8)		28 123 (4.6)	28 140 (4.6)	
2018	24 606 (4.0)	73 284 (5.2)		28 123 (4.0)	24 618 (4.0)	
2019	11 774 (1.9)	34 864 (2.5)		11 774 (1.9)	11 776 (1.9)	
2020	4558 (0.7)	14 273 (1.0)		4558 (0.7)	4555 (0.7)	
**Healthcare utilization**						
Number of distinct generics, mean (SD)	9.44 (5.86)	9.15 (5.78)	0.049	9.41 (5.85)	9.43 (5.86)	0.002
Number of physician visits, mean (SD)	5.33 (4.31)	5.44 (4.34)	0.026	5.33 (4.31)	5.34 (4.27)	0.003
Number of hospitalizations, mean, (SD)	0.34 (0.77)	0.37 (0.85)	0.033	0.34 (0.77)	0.34 (0.76)	0.001
Number of hospital days for hospitalizations, mean (SD)	1.95 (6.46)	2.22 (7.54)	0.038	1.95 (6.46)	1.95 (6.42)	<0.001
Hospitalization in 30 days before index date	118 630 (19.2)	285 570 (20.1)	0.024	117 942 (19.2)	117 540 (19.1)	0.002
**Comorbidities**						
Combined comorbidity score, mean (SD)	0.85 (1.82)	0.97 (2.01)	0.063	0.85 (1.82)	0.85 (1.83)	0.001
Alcohol abuse/dependence	7534 (1.2)	17 995 (1.3)	0.005	7454 (1.2)	7478 (1.2)	<0.001
Anxiety	145 133 (23.5)	368 942 (26.0)	0.059	144 172 (23.5)	144 379 (23.5)	0.001
Back and neck pain	213 302 (34.5)	480 401 (33.9)	0.013	211 468 (34.4)	211 825 (34.5)	0.001

© 2022 Yunusa I et al. *JAMA Network Open.*

Bipolar disorder	29 533 (4.8)	55 819 (3.9)	0.041	29 156 (4.7)	29 530 (4.8)	0.001
Back pain- without radiculopathy	193 753 (31.3)	433 119 (30.5)	0.017	191 986 (31.2)	192 272 (31.3)	0.001
Back pain- with radiculopathy	56 462 (9.1)	127 185 (9.0)	0.006	56 116 (9.1)	56 221 (9.1)	0.001
Bone fracture	14 378 (2.3)	41 851 (2.9)	0.039	14 377 (2.3)	14 385 (2.3)	<0.001
Cancer	50 887 (8.2)	128 400 (9.1)	0.029	50 656 (8.2)	50 570 (8.2)	<0.001
COPD/Asthma/Oxygen Use	92 373 (14.9)	207 671 (14.6)	0.008	91 458 (14.9)	91 499 (14.9)	<0.001
Dementia	11 966 (1.9)	42 181 (3.0)	0.067	11 954 (1.9)	11 957 (1.9)	<0.001
Dental Pain	12 352 (2.0)	23 015 (1.6)	0.028	12 006 (2.0)	12 052 (2.0)	0.001
Depression	178 850 (28.9)	434 992 (30.7)	0.038	177 706 (28.9)	178 363 (29.0)	0.002
Diabetes	94 277 (15.2)	215 056 (15.2)	0.002	93 587 (15.2)	93 498 (15.2)	<0.001
Diabetic neuropathy	14 476 (2.3)	35 465 (2.5)	0.01	14 399 (2.3)	14 413 (2.3)	<0.001
Epilepsy or convulsions	15 649 (2.5)	35 218 (2.5)	0.003	15 404 (2.5)	15 459 (2.5)	0.001
Fibromyalgia	42 235 (6.8)	87 819 (6.2)	0.026	41 791 (6.8)	42 011 (6.8)	0.001
Headache	87 642 (14.2)	195 453 (13.8)	0.011	86 759 (14.1)	87 054 (14.2)	0.001
Intentional Self-Harm	1684 (0.3)	4036 (0.3)	0.002	1676 (0.3)	1680 (0.3)	<0.001
Liver Disease	37 041 (6.0)	89 621 (6.3)	0.014	36 761 (6.0)	36 812 (6.0)	0.001
Musculoskeletal Injury	76 285 (12.3)	175 443 (12.4)	0.001	75 766 (12.3)	75 906 (12.4)	0.001
Opioid Dependence/Abuse	7582 (1.2)	14 835 (1.0)	0.017	7445 (1.2)	7466 (1.2)	<0.001
Osteoarthritis	107 848 (17.4)	248 164 (17.5)	0.002	107 272 (17.5)	107 210 (17.5)	<0.001
Other arthritis, arthropathies and musculoskeletal pain	283 302 (45.8)	659 259 (46.5)	0.014	281 350 (45.8)	281 579 (45.8)	0.001
Other neuropathic pain	109 214 (17.6)	249 634 (17.6)	0.001	108 505 (17.7)	108 727 (2.3)	0.001
Other drug abuse or dependence	14 612 (2.4)	28 573 (2.0)	0.024	14 345 (2.3)	14 410 (2.3)	0.001
Postherpetic neuralgia	830 (0.1)	2162 (0.2)	0.005	823 (0.1)	816 (0.1)	<0.001
Previous overdose excl index	891 (0.1)	1699 (0.1)	0.007	870 (0.1)	868 (0.1)	<0.001
Psychosis	14 132 (2.3)	29 951 (2.1)	0.012	13 856 (2.3)	13 931 (2.3)	0.001
Renal Dysfunction	38 050 (6.1)	99 132 (7.0)	0.034	37 934 (6.2)	37 890 (6.2)	<0.001
Rheumatoid arthritis	12 434 (2.0)	27 110 (1.9)	0.007	12 319 (2.0)	12 342 (2.0)	<0.001
Tobacco	71 124 (11.5)	167 210 (11.8)	0.009	70 807 (11.5)	70 901 (11.5)	0.001

© 2022 Yunusa I et al. *JAMA Network Open.*

Urinary calculus	31 176 (5.0)	75 031 (5.3)	0.011	31 026 (5.1)	31 100 (5.1)	0.001
**Opioid-related medication use**						
Total oxycodone MME on index date, mean (SD)	423.94(554.70)	413.14(539.64)	0.02	429.77 (595.47)	430.12 (598.12)	0.001
Total Non-oxycodone MME on index date, mean (SD)	25.64(136.46)	23.89(130.97)	0.013	28.52 (160.72)	28.56 (160.60)	<0.001
Total MME 60 days prior to index date mean, (SD)	559.93(1464.62)	484.04(1364.58)	0.054	583.96 (1644.74)	584.43 (1655.84)	<0.001
Total MME 180 days prior to index date, mean, (SD)	1571.82(4243.76)	1357.44(3947.98)	0.052	1633.61 (4723.77)	1635.05 (4759.39)	<0.001
Codeine 60 pre-index date	29 403 (4.8)	58 255 (4.1)	0.031	28 711 (4.7)	28 705 (4.7)	<0.001
Codeine 180 pre-index date	56 963 (9.2)	117 323 (8.3)	0.033	55 856 (9.1)	55 875 (9.1)	<0.001
Codeine on index date	1951 (0.3)	3893 (0.3)	0.008	1901 (0.3)	1899 (0.3)	0.001
Hydrocodone 60 pre-index date	180 077 (29.1)	372 804 (26.3)	0.063	178 246 (29.0)	178 382 (29.0)	0.001
Hydrocodone 180 pre-index date	240 512 (38.9)	508 511 (35.8)	0.063	238 193 (38.8)	238 387 (38.8)	0.001
Hydrocodone on index date	14 584 (2.4)	30 739 (2.2)	0.013	14 433 (2.3)	14 459 (2.4)	<0.001
Hydromorphone 60 pre-index date	4995 (0.8)	11 407 (0.8)	<0.001	4969 (0.8)	4986 (0.8)	<0.001
Hydromorphone 180 pre-index date	7489 (1.2)	17 242 (1.2)	<0.001	7452 (1.2)	7481 (1.2)	<0.001
Hydromorphone on index date	1634 (0.3)	3757 (0.3)	<0.001	1629 (0.3)	1638 (0.3)	<0.001
Levorphanol 60 pre-index date	15 (0.0)	49 (0.0)	0.002	14 (0.0)	14 (0.0)	<0.001
Levorphanol 180 pre-index date	25 (0.0)	66 (0.0)	0.001	24 (0.0)	25 (0.0)	<0.001
Levorphanol on index date	<11	<11	<0.001	<11	<11	<0.001
Meperidine 60 pre-index date	2180 (0.4)	4025 (0.3)	0.012	2145 (0.3)	2161 (0.4)	<0.001
Meperidine 180 pre-index	3881 (0.6)	7061 (0.5)	0.017	3821 (0.6)	3850 (0.6)	0.001
Meperidine on index date	573 (0.1)	1231 (0.1)	0.002	564 (0.1)	566 (0.1)	<0.001
Methadone 60 pre-index date	4063 (0.7)	6895 (0.5)	0.023	3978 (0.6)	3983 (0.6)	<0.001
Methadone 180 pre-index date	5183 (0.8)	8818 (0.6)	0.025	5074 (0.8)	5089 (0.8)	<0.001
Methadone on index date	994 (0.2)	1862 (0.1)	0.008	976 (0.2)	977 (0.2)	<0.001
Morphine 60 pre-index date	8704 (1.4)	17 355 (1.2)	0.016	8599 (1.4)	8613 (1.4)	<0.001
Morphine 180 pre-index date	11 410 (1.8)	22 914 (1.6)	0.018	11 275 (1.8)	11 289 (1.8)	<0.001
Morphine on index date	3481 (0.6)	7527 (0.5)	0.004	3449 (0.6)	3451 (0.6)	<0.001

© 2022 Yunusa I et al. *JAMA Network Open.*

Naloxone 60 pre-index date	2306 (0.4)	4728 (0.3)	0.007	2286 (0.4)	2303 (0.4)	<0.001
Naloxone 180 pre-index date	3380 (0.5)	6927 (0.5)	0.008	3349 (0.5)	3375 (0.5)	0.001
Naloxone on index date	242 (0.0)	618 (0.0)	0.002	240 (0.0)	241 (0.00)	<0.001
Oxymorphone 60 pre-index date	842 (0.1)	2153 (0.2)	0.004	842 (0.1)	843 (0.1)	<0.001
Oxymorphone 180 pre-index date	1121 (0.2)	2854 (0.2)	0.005	1121 (0.2)	1123 (0.2)	<0.001
Oxymorphone on index date	174 (0.0)	422 (0.0)	0.001	174 (0.0)	177 (0.0)	<0.001
Pentazocine 60 pre-index date	784 (0.1)	1237 (0.1)	0.012	759 (0.1)	768 (0.1)	<0.001
Pentazocine 180 pre-index	1317 (0.2)	2140 (0.2)	0.015	1279 (0.2)	1290 (0.2)	<0.001
Pentazocine on index date	28 (0.0)	55 (0.0)	0.001	26 (0.0)	27 (0.0)	<0.001
Propoxyphene 60 pre-index date	23 577 (3.8)	38 481 (2.7)	0.062	23 014 (3.7)	22 953 (3.7)	0.001
Propoxyphene 180 pre-index date	38 909 (6.3)	65 446 (4.6)	0.074	38 074 (6.2)	38 017 (6.2)	<0.001
Propoxyphene on index date	1837 (0.3)	3319 (0.2)	0.012	1810 (0.3)	1802 (0.3)	<0.001
Tramadol 60 pre-index date	43 099 (7.0)	104 214 (7.3)	0.015	42 787 (7.0)	42 932 (7.0)	0.001
Tramadol 180 pre-index date	69 369 (11.2)	163 506 (11.5)	0.01	68 807 (11.2)	69 048 (11.2)	0.001
Tramadol on index date	5293 (0.9)	13 422 (0.9)	0.01	5272 (0.9)	5293 (0.9)	<0.001
Fentanyl 60 pre-index date	8994 (1.5)	18 567 (1.3)	0.012	8893 (1.4)	8912 (1.5)	<0.001
Fentanyl 180 pre-index date	11 580 (1.9)	24 066 (1.7)	0.013	11 455 (1.9)	11 483 (1.9)	<0.001
Fentanyl on index date	3513 (0.6)	7661 (0.5)	0.004	3492 (0.6)	3495 (0.6)	<0.001
Tapentadol 60 pre-index date	985 (0.2)	2513 (0.2)	0.004	985 (0.2)	987 (0.2)	<0.001
Tapentadol 180 pre-index date	1474 (0.2)	3856 (0.3)	0.007	1474 (0.2)	1481 (0.2)	<0.001
Tapentadol on index date	138 (0.0)	369 (0.0)	0.002	138 (0.0)	138 (0.0)	<0.001
Dihydrocodeine 60 pre-index date	450 (0.1)	753 (0.1)	0.008	439 (0.1)	440 (0.1)	<0.001
Dihydrocodeine 180 pre-index date	1009 (0.2)	1565 (0.1)	0.014	971 (0.2)	975 (0.2)	<0.001
Dihydrocodeine on index date	26 (0.0)	18 (0.0)	0.006	16 (0.0)	16 (0.0)	<0.001
Butorphanol 60 pre-index date	761 (0.1)	1213 (0.1)	0.012	728 (0.1)	742 (0.1)	0.001
Butorphanol 180 pre-index date	1025 (0.2)	1693 (0.1)	0.012	985 (0.2)	1003 (0.2)	0.001
Butorphanol on index date	83 (0.0)	110 (0.0)	0.006	73 (0.0)	75 (0.0)	<0.001
Buprenorphine 60 pre-index date	2358 (0.4)	5279 (0.4)	0.001	2356 (0.4)	2365 (0.4)	<0.001
Buprenorphine 180 pre-index date	3210 (0.5)	7179 (0.5)	0.002	3208 (0.5)	3221 (0.5)	<0.001

© 2022 Yunusa I et al. *JAMA Network Open.*

Buprenorphine on index date	132 (0.0)	344 (0.0)	0.002	131 (0.0)	131 (0.0)	<0.001
**Other Prior medications**						
SSRI 180 pre-index date	606 623 (98.0)	1 379 543 (97.2)	0.052	602 275 (98.0)	602 254 (98.0)	<0.001
Other antidepressants 180 plus index date	162 188 (26.2)	344 128 (24.3)	0.045	160 391 (26.1)	161 212 (26.2)	0.003
Benzodiazepines 180 plus index date	229 768 (37.1)	508 449 (35.8)	0.027	227 523 (37.0)	228 132 (37.1)	0.002
Muscle Relaxants 180 plus index date	140 775 (22.8)	299 501 (21.1)	0.04	139 431 (22.7)	139 735 (22.7)	0.001
Other Sedatives-Hypnotics 180 plus index date	83 542 (13.5)	198 287 (14.0)	0.014	83 046 (13.5)	83 354 (13.6)	0.001
NSAID 180 plus index date	238 226 (38.5)	522 889 (36.9)	0.034	235 713 (38.4)	235 789 (38.4)	<0.001
Lithium 180 plus index date	5580 (0.9)	9210 (0.6)	0.029	5436 (0.9)	5516 (0.9)	0.001
Atypical antipsychotics 180 plus index date	54 791 (8.9)	105 438 (7.4)	0.052	54 107 (8.8)	54 632 (8.9)	0.003
Typical antipsychotics 180 plus index	4717 (0.8)	8682 (0.6)	0.018	4549 (0.7)	4577 (0.7)	0.001
Barbiturates 180 plus index date	3311 (0.5)	5700 (0.4)	0.02	3233 (0.5)	3235 (0.5)	<0.001
Agents for dementia 180 plus index date	5786 (0.9)	22 194 (1.6)	0.057	5782 (0.9)	5783 (0.9)	<0.001
Anticonvulsants 180 plus index date	63 887 (10.3)	136 013 (9.6)	0.025	63 305 (10.3)	63 908 (10.4)	0.003
Gabapentinoids 180 plus index	81 383 (13.2)	183 276 (12.9)	0.007	80 791 (13.1)	81 117 (13.2)	0.002
Triptans 180 plus index date	29 940 (4.8)	62 457 (4.4)	0.021	29 667 (4.8)	29 850 (4.9)	0.001
CYP 3A4 inhibitors overlapping index date	52 695 (8.5)	119 390 (8.4)	0.004	52 234 (8.5)	52 192 (8.5)	<0.001
CYP 3A4 inducers overlapping index date	29 604 (4.8)	62 288 (4.4)	0.019	29 329 (4.8)	29 583 (4.8)	0.002
CYP 2D6 inhibitors overlapping index date	6769 (1.1)	16 020 (1.1)	0.003	6707 (1.1)	6674 (1.1)	<0.001
CYP 2D6 inducers overlapping index date	9539 (1.5)	18 944 (1.3)	0.017	9355 (1.5)	9334 (1.5)	<0.001

Abbreviations: SSRI, selective serotonin reuptake inhibitors; SD, standard deviation; COPD, chronic obstructive pulmonary disease; MME, morphine milligram equivalent; Std. diff., standardized difference; NSAID, Nonsteroidal anti-inflammatory drugs; CYP, cytochrome P450

[a]Counts less than 11 were suppressed per Centers for Medicare & Medicaid Services policy.

[b]Standardized differences greater than 0.1 indicate lack of balance.

© 2022 Yunusa I et al. *JAMA Network Open.*

eTable 4. Patient Baseline Characteristics Before and After Propensity Score Weighting for MarketScan Database

Patient characteristic	SSRI Exposure groups					
	Unweighted			Weighted		
	CPY2D6-inhibiting SSRI	Other SSRI	[a]Std. diff.	CPY2D6-inhibiting SSRI	Other SSRI	[a]Std. diff.
**Number of patients**	337 586	815 898		337 427	337 401	
**Demographics**						
Age, mean (SD), y	50.53 (14.14)	49.73 (14.97)	0.055	50.48 (14.22)	50.53 (14.14)	
Age category, y			0.112			0.002
18-24	14 567 (4.3)	40 707 (5.0)		14 566 (4.3)	14 648 (4.3)	
25-34	32 544 (9.6)	97 345 (11.9)		32 543 (9.6)	32 533 (9.6)	
35-44	62 244 (18.4)	158 343 (19.4)		62 234 (18.4)	62 266 (18.5)	
45-54	90 230 (26.7)	203 100 (24.9)		90 177 (26.7)	90 334 (26.8)	
55-64	93 384 (27.7)	207 775 (25.5)		93 320 (27.7)	93 183 (27.6)	
65-74	28 490 (8.4)	61 366 (7.5)		28 458 (8.4)	28 387 (8.4)	
75 and above	16 127 (4.8)	47 262 (5.8)		16 126 (4.8)	16 046 (4.8)	
Sex			0.039			<0.001
Female	247 317 (73.3)	583 404 (71.5)		247 172 (73.3)	247 086 (73.2)	
Region			0.078			0.002
Midwest	70 632 (20.9)	174 861 (21.4)		70 617 (20.9)	70 628 (20.9)	
Northeast	60 709 (18.0)	158 788 (19.5)		60 704 (18.0)	60 722 (18.0)	
South	142 241 (42.1)	349 748 (42.9)		142 212 (42.1)	142 432 (42.2)	
West	61 734 (18.3)	126 555 (15.5)		61 622 (18.3)	61 345 (18.2)	
Unknown	2270 (0.7)	5946 (0.7)		2269 (0.7)	2272 (0.7)	
Calendar year of index date			0.223			0.001
2003	9094 (2.7)	11 414 (1.4)		9005 (2.7)	9033 (2.7)	
2004	18 813 (5.6)	26 238 (3.2)		18 756 (5.6)	18 755 (5.6)	

© 2022 Yunusa I et al. *JAMA Network Open.*

2005	20 685 (6.1)	32 396 (4.0)		20 672 (6.1)	20 651 (6.1)	
2006	18 419 (5.5)	33 387 (4.1)		18 418 (5.5)	18 409 (5.5)	
2007	18 909 (5.6)	39 083 (4.8)		18 908 (5.6)	18 874 (5.6)	
2008	22 979 (6.8)	50 966 (6.2)		22 978 (6.8)	22 946 (6.8)	
2009	27 271 (8.1)	66 065 (8.1)		27 270 (8.1)	27 271 (8.1)	
2010	27 690 (8.2)	69 780 (8.6)		27 690 (8.2)	27 690 (8.2)	
2011	30 202 (8.9)	78 727 (9.6)		30 202 (9.0)	30 191 (8.9)	
2012	29 750 (8.8)	80 305 (9.8)		29 750 (8.8)	29 767 (8.8)	
2013	23 745 (7.0)	66 233 (8.1)		23 745 (7.0)	23 738 (7.0)	
2014	23 786 (7.0)	67 929 (8.3)		23 786 (7.0)	23 791 (7.1)	
2015	19 278 (5.7)	54 726 (6.7)		19 278 (5.7)	19 282 (5.7)	
2016	18 383 (5.4)	53 314 (6.5)		18 383 (5.4)	18 403 (5.5)	
2017	15 497 (4.6)	45 935 (5.6)		15 497 (4.6)	15 504 (4.6)	
2018	13 085 (3.9)	39 400 (4.8)		13 085 (3.9)	13 090 (3.9)	
**Healthcare utilization**						
Number of distinct generics, mean (SD)	8.78 (5.30)	8.60 (5.31)	0.035	8.78 (5.30)	8.80 (5.35)	0.003
Number of physician visits, mean (SD)	5.47 (4.23)	5.53 (4.27)	0.014	5.47 (4.23)	5.48 (4.19)	0.003
Number of hospitalizations, mean, (SD)	0.28 (0.56)	0.30 (0.58)	0.033	0.28 (0.56)	0.28 (0.55)	0.001
Number of hospital days for hospitalizations, mean (SD)	1.53 (5.20)	1.78 (6.20)	0.043	1.53 (5.20)	1.53 (5.07)	<0.001
Hospitalization in 30 days before index date	61 814 (18.3)	155 894 (19.1)	0.02	61 793 (18.3)	61 542 (18.2)	0.002
**Comorbidities**						
Combined comorbidity score, mean (SD)	0.63 (1.58)	0.72 (1.72)	0.055	0.63 (1.58)	0.63 (1.59)	0.001
Alcohol abuse/dependence	2503 (0.7)	6681 (0.8)	0.009	2502 (0.7)	2517 (0.7)	0.001
Anxiety	69 472 (20.6)	189 609 (23.2)	0.064	69 461 (20.6)	69 571 (20.6)	0.001
Back and neck pain	106 346 (31.5)	254 081 (31.1)	0.008	106 279 (31.5)	106 466 (31.6)	0.001
Bipolar disorder	11 026 (3.3)	22 275 (2.7)	0.031	10 996 (3.3)	11 176 (3.3)	0.003
Back pain- without radiculopathy	96 314 (28.5)	229 388 (28.1)	0.009	96 249 (28.5)	96 399 (28.6)	0.001
Back pain- with radiculopathy	28 892 (8.6)	68 356 (8.4)	0.006	28 873 (8.6)	28 890 (8.6)	<0.001
Bone fracture	5677 (1.7)	16 319 (2.0)	0.024	5677 (1.7)	5680 (1.7)	<0.001

© 2022 Yunusa I et al. *JAMA Network Open.*

Cancer	27 536 (8.2)	71 726 (8.8)	0.023	27 532 (8.2)	27 456 (8.1)	0.001
COPD/Asthma/Oxygen Use	38 415 (11.4)	92 581 (11.3)	0.001	38 387 (11.4)	38 413 (11.4)	<0.001
Dementia	4638 (1.4)	16 214 (2.0)	0.048	4637 (1.4)	4635 (1.4)	<0.001
Dental Pain	3143 (0.9)	7204 (0.9)	0.005	3140 (0.9)	3136 (0.9)	<0.001
Depression	81 037 (24.0)	213 354 (26.1)	0.049	81 017 (24.0)	81 321 (24.1)	0.002
Diabetes	43 013 (12.7)	102 250 (12.5)	0.006	42 992 (12.7)	42 948 (12.7)	<0.001
Diabetic neuropathy	5377 (1.6)	13 392 (1.6)	0.004	5374 (1.6)	5374 (1.6)	<0.001
Epilepsy or convulsions	5105 (1.5)	13 595 (1.7)	0.012	5103 (1.5)	5123 (1.5)	0.001
Fibromyalgia	19 780 (5.9)	44 401 (5.4)	0.018	19 757 (5.9)	19 859 (5.9)	0.001
Headache	41407 (12.3)	100 030 (12.3)	<0.001	41 379 (12.3)	41 531 (12.3)	0.001
Intentional Self-Harm	500 (0.1)	1306 (0.2)	0.003	498 (0.1)	501 (0.1)	<0.001
Liver Disease	16 006 (4.7)	41 896 (5.1)	0.018	16 001 (4.7)	16 027 (4.8)	<0.001
Musculoskeletal Injury	42 177 (12.5)	103 094 (12.6)	0.004	42 165 (12.5)	42 228 (12.5)	0.001
Opioid Dependence/Abuse	2108 (0.6)	4599 (0.6)	0.008	2103 (0.6)	2113 (0.6)	<0.001
Osteoarthritis	55 026 (16.3)	129 497 (15.9)	0.012	54 995 (16.3)	54 943 (16.3)	<0.001
Other arthritis arthropathies and musculoskeletal pain	147 292 (43.6)	360 486 (44.2)	0.011	147 232 (43.6)	147 364 (43.7)	0.001
Other neuropathic pain	56 062 (16.6)	134 226 (16.5)	0.004	56 031 (16.6)	56 139 (16.6)	0.001
Other drug abuse or dependence	3775 (1.1)	8035 (1.0)	0.013	3763 (1.1)	3796 (1.1)	0.001
Postherpetic neuralgia	435 (0.1)	1119 (0.1)	0.002	434 (0.1)	432 (0.1)	<0.001
Previous overdose excl index	253 (0.1)	516 (0.1)	0.004	252 (0.1)	252 (0.1)	<0.001
Psychosis	3387 (1.0)	8152 (1.0)	<0.001	3381 (1.0)	3413 (1.0)	0.001
Renal dysfunction	16 689 (4.9)	44 481 (5.5)	0.023	16 685 (4.9)	16 663 (4.9)	<0.001
Rheumatoid arthritis	5929 (1.8)	13 395 (1.6)	0.009	5922 (1.8)	5930 (1.8)	<0.001
Tobacco	25 459 (7.5)	62 519 (7.7)	0.005	25 447 (7.5)	25 445 (7.5)	<0.001
Urinary calculus	17 131 (5.1)	42 972 (5.3)	0.009	17 127 (5.1)	17 168 (5.1)	0.001
**Opioid-related medication use**						
Total oxycodone MME on index date, mean (SD)	449.08(647.46)	442.76 (643.94)	0.01	448.94 (647.25)	449.47 (651.59)	<0.001
Total Non-oxycodone MME on index date, mean (SD)	28.03 (152.94)	25.90 (145.98)	0.014	28.00 (152.86)	28.03 (152.78)	<0.001
Total MME 60 days prior to index date, mean (SD)	546.03(1586.99)	479.24 (1491.06)	0.043	545.25(1585.66)	546.00(1602.84)	<0.001

© 2022 Yunusa I et al. *JAMA Network Open.*

Total MME 180 days prior to index date, mean (SD)	1521.37(4547.51)	1329.10 (4245.18)	0.044	1519.00(4543.11)	1521.57(4587.60)	0.001
Codeine 60 pre-index date	13 925 (4.1)	30 802 (3.8)	0.018	13 903 (4.1)	13 889 (4.1)	<0.001
Codeine 180 pre-index date	27 951 (8.3)	63 952 (7.8)	0.016	27 919 (8.3)	27 905 (8.3)	<0.001
Codeine on index date	1013 (0.3)	2251 (0.3)	0.005	1011 (0.3)	1007 (0.3)	<0.001
Hydrocodone 60 pre-index date	92 391 (27.4)	204 423 (25.1)	0.053	92 287 (27.4)	92 339 (27.4)	<0.001
Hydrocodone 180 pre-index date	125 056 (37.0)	283 227 (34.7)	0.049	124 940 (37.0)	125 052 (37.1)	0.001
Hydrocodone on index date	8498 (2.5)	18 938 (2.3)	0.013	8487 (2.5)	8504 (2.5)	<0.001
Hydromorphone 60 pre-index date	2435 (0.7)	5780 (0.7)	0.002	2433 (0.7)	2446 (0.7)	<0.001
Hydromorphone 180 pre-index date	3615 (1.1)	8755 (1.1)	<0.001	3612 (1.1)	3635 (1.1)	0.001
Hydromorphone on index date	888 (0.3)	2165 (0.3)	<0.001	887 (0.3)	895 (0.3)	<0.001
Levorphanol 60 pre-index date	3 (0.0)	27 (0.0)	0.005	3 (0.0)	3 (0.0)	<0.001
Levorphanol 180 pre-index date	8 (0.0)	35 (0.0)	0.003	8 (0.0)	8 (0.0)	<0.001
Levorphanol on index date	1 (0.0)	5 (0.0)	0.001	1 (0.0)	1 (0.0)	0.001
Meperidine 60 pre-index date	1210 (0.4)	2404 (0.3)	0.011	1207 (0.4)	1216 (0.4)	<0.001
Meperidine 180 pre-index	2196 (0.7)	4242 (0.5)	0.017	2191 (0.6)	2212 (0.7)	0.001
Meperidine on index date	366 (0.1)	839 (0.1)	0.002	365 (0.1)	367 (0.1)	<0.001
Methadone 60 pre-index date	1491 (0.4)	2727 (0.3)	0.017	1483 (0.4)	1478 (0.4)	<0.001
Methadone 180 pre-index date	1902 (0.6)	3465 (0.4)	0.02	1890 (0.6)	1894 (0.6)	<0.001
Methadone on index date	357 (0.1)	763 (0.1)	0.004	355 (0.1)	358 (0.1)	<0.001
Morphine 60 pre-index date	3584 (1.1)	7582 (0.9)	0.013	3574 (1.1)	3579 (1.1)	<0.001
Morphine 180 pre-index date	4734 (1.4)	10 075 (1.2)	0.015	4720 (1.4)	4726 (1.4)	<0.001
Morphine on index date	1583 (0.5)	3677 (0.5)	0.003	1580.2 (0.5)	1576 (0.5)	<0.001
Naloxone 60 pre-index date	1111 (0.3)	2422 (0.3)	0.006	1108 (0.3)	1117 (0.3)	<0.001
Naloxone 180 pre-index date	1579 (0.5)	3383 (0.4)	0.008	1576 (0.5)	1589 (0.5)	0.001
Naloxone on index date	63 (0.0)	160 (0.0)	0.001	63 (0.0)	63 (0.0)	<0.001
Oxymorphone 60 pre-index date	502 (0.1)	1233 (0.2)	0.001	502 (0.1)	502 (0.1)	<0.001
Oxymorphone 180 pre-index date	649 (0.2)	1630 (0.2)	0.002	649 (0.2)	649 (0.2)	<0.001
Oxymorphone on index date	107 (0.0)	243 (0.0)	0.001	107 (0.0)	108 (0.0)	<0.001
Pentazocine 60 pre-index date	326 (0.1)	588 (0.1)	0.008	323 (0.1)	330 (0.1)	0.001

© 2022 Yunusa I et al. *JAMA Network Open.*

Pentazocine 180 pre-index	523 (0.2)	975 (0.1)	0.01	520 (0.2)	526 (0.2)	<0.001
Pentazocine on index date	12 (0.0)	24 (0.0)	0.001	12.0 (0.0)	12 (0.0)	<0.001
Propoxyphene 60 pre-index date	10 760 (3.2)	19 400 (2.4)	0.049	10 730 (3.2)	10 698 (3.2)	0.001
Propoxyphene 180 pre-index date	17 843 (5.3)	33 071 (4.1)	0.058	17 803 (5.3)	17 774 (5.3)	<0.001
Propoxyphene on index date	1013 (0.3)	1954 (0.2)	0.012	1012.3 (0.3)	1005 (0.3)	<0.001
Tramadol 60 pre-index date	20 580 (6.1)	53 221 (6.5)	0.018	20 576 (6.1)	20 701 (6.1)	0.002
Tramadol 180 pre-index date	33 151 (9.8)	84 146 (10.3)	0.016	33 141 (9.8)	33 307 (9.9)	0.002
Tramadol on index date	2691 (0.8)	7058 (0.9)	0.007	2690 (0.8)	2703 (0.8)	<0.001
Fentanyl 60 pre-index date	4117 (1.2)	9014 (1.1)	0.011	4111 (1.2)	4130 (1.2)	0.001
Fentanyl 180 pre-index date	5240 (1.6)	11 485 (1.4)	0.012	5232 (1.6)	5251 (1.6)	<0.001
Fentanyl on index date	1630 (0.5)	3669 (0.4)	0.005	1627 (0.5)	1625 (0.5)	<0.001
Tapentadol 60 pre-index date	655 (0.2)	1673 (0.2)	0.002	655 (0.2)	656 (0.2)	<0.001
Tapentadol 180 pre-index date	973 (0.3)	2551 (0.3)	0.004	973 (0.3)	975 (0.3)	<0.001
Tapentadol on index date	108 (0.0)	263 (0.0)	<0.001	108 (0.0)	107 (0.0)	<0.001
Dihydrocodeine 60 pre-index date	168 (0.0)	314 (0.0)	0.005	167 (0.0)	170 (0.1)	<0.001
Dihydrocodeine 180 pre-index date	389 (0.1)	678 (0.1)	0.01	385 (0.1)	389 (0.1)	<0.001
Dihydrocodeine on index date	7 (0.0)	8 (0.0)	0.003	6 (0.0)	6 (0.0)	<0.001
Butorphanol 60 pre-index date	321 (0.1)	600 (0.1)	0.007	318 (0.1)	326 (0.1)	0.001
Butorphanol 180 pre-index date	438 (0.1)	845 (0.1)	0.008	435 (0.1)	447 (0.1)	0.001
Butorphanol on index date	40 (0.0)	52 (0.0)	0.006	37 (0.0)	39 (0.0)	<0.001
Buprenorphine 60 pre-index date	1249 (0.4)	2944 (0.4)	0.002	1248 (0.4)	1256 (0.4)	<0.001
Buprenorphine 180 pre-index date	1740 (0.5)	3992 (0.5)	0.004	1739 (0.5)	1748 (0.5)	<0.001
Buprenorphine on index date	70 (0.0)	197 (0.0)	0.002	70 (0.0)	70 (0.0)	<0.001
**Other prior medications**						
SSRI 180 pre-index date	332 232 (98.4)	795 717 (97.5)	0.063	332 073 (98.4)	332 059 (98.4)	<0.001
Other antidepressants 180 plus index date	78 967 (23.4)	177 802 (21.8)	0.038	78 875 (23.4)	79 325 (23.5)	0.003
Benzodiazepines 180 plus index date	123 598 (36.6)	292 390 (35.8)	0.016	123 504 (36.6)	123 876 (36.7)	0.002
Muscle Relaxants 180 plus index date	72 439 (21.5)	164 465 (20.2)	0.032	72 364 (21.4)	72 565 (21.5)	0.001
Other Sedatives-Hypnotics 180 plus index date	45 481 (13.5)	115 265 (14.1)	0.019	45 473 (13.5)	45 653 (13.5)	0.002

© 2022 Yunusa I et al. *JAMA Network Open.*

NSAID 180 plus index date	124 430 (36.9)	294 879 (36.1)	0.015	124 354 (36.9)	124 379 (36.9)	<0.001
Lithium 180 plus index date	2335 (0.7)	4109 (0.5)	0.024	2318 (0.7)	2363 (0.7)	0.002
Atypical antipsychotics 180 plus index date	19 890 (5.9)	40 448 (5.0)	0.041	19 839 (5.9)	20 137 (6.0)	0.004
Typical antipsychotics 180 plus index	1283 (0.4)	2581 (0.3)	0.011	1276 (0.4)	1293 (0.4)	0.001
Barbiturates 180 plus index date	1746 (0.5)	3282 (0.4)	0.017	1739 (0.5)	1740 (0.5)	<0.001
Agents for dementia 180 plus index date	2688 (0.8)	10 368 (1.3)	0.047	2687 (0.8)	2692 (0.8)	<0.001
Anticonvulsants 180 plus index date	29 802 (8.8)	67 662 (8.3)	0.019	29 761 (8.8)	30 091 (8.9)	0.003
Gabapentinoids 180 plus index	37 673 (11.2)	88 665 (10.9)	0.009	37 644 (11.2)	37 816 (11.2)	0.002
Triptans 180 plus index date	16 793 (5.0)	37 318 (4.6)	0.019	16 774 (5.0)	16 873 (5.0)	0.001
CYP 3A4 inhibitors overlapping index date	26 944 (8.0)	64 315 (7.9)	0.004	26 925 (8.0)	26 933 (8.0)	<0.001
CYP 3A4 inducers overlapping index date	14 057 (4.2)	31 810 (3.9)	0.013	14 039 (4.2)	14 174 (4.2)	0.002
CYP 2D6 inhibitors overlapping index date	2704 (0.8)	6806 (0.8)	0.004	2701 (0.8)	2685 (0.8)	0.001
CYP 2D6 inducers overlapping index date	3223 (1.0)	7084 (0.9)	0.009	3217 (1.0)	3227 (1.0)	<0.001

Abbreviations: SSRI, selective serotonin reuptake inhibitors; SD, standard deviation; COPD, chronic obstructive pulmonary disease; MME, morphine milligram equivalent; Std. diff., standardized difference; NSAID, Nonsteroidal anti-inflammatory drugs; CYP, cytochrome P450

[a]Standardized differences greater than 0.1 indicate lack of balance.

© 2022 Yunusa I et al. *JAMA Network Open.*

eTable 5. Patient Baseline Characteristics Before and After Propensity Score Weighting for MAX Database

Patient characteristic	SSRI Exposure groups[a]					
	Unweighted			Weighted		
	CPY2D6-inhibiting SSRI	Other SSRI	[b]Std. diff.	CPY2D6-inhibiting SSRI	Other SSRI	[b]Std. diff.
**Number of patients**	98 192	159 636		93 998	93 978	
**Demographics**						
Age, mean (SD), y	41.24 (11.58)	40.58 (11.88)	0.056	41.10 (11.62)	41.04 (11.63)	0.005
Age category, y			0.07			0.002
18-24	9407 (9.6)	18 087 (11.3)		9275 (9.9)	9274 (9.9)	
25-34	20 486 (20.9)	35 148 (22.0)		19 904 (21.2)	19 951 (21.2)	
35-44	26 448 (26.9)	41 173 (25.8)		25 181 (26.8)	25 197 (26.8)	
45-54	27 895 (28.4)	42 920 (26.9)		26 395 (28.1)	26 375 (28.1)	
55-64	13 956 (14.2)	22 308 (14.0)		13 241 (14.1)	13 178 (14.0)	
Sex			0.049			<0.001
Female	76 729 (78.1)	121 521 (76.1)		73 209 (77.9)	73 190 (77.9)	
Region			0.099			0.004
Midwest	23 471 (23.9)	40 783 (25.5)		22 681 (24.1)	22 797 (24.3)	
Northeast	22 962 (23.4)	40 547 (25.4)		22 235 (23.7)	22 235 (23.7)	
South	40 189 (40.9)	63 754 (39.9)		38 704 (41.2)	38 544 (41.0)	
West	11 491 (11.7)	14 420 (9.0)		10 300 (11.0)	10 326 (11.0)	
Unknown	79 (0.1)	132 (0.1)		75 (0.1)	75 (0.1)	
Calendar year of index date			0.365			0.001
2000	5042 (5.1)	3726 (2.3)		3702 (3.9)	3704 (3.9)	
2001	10 068 (10.3)	8196 (5.1)		8091 (8.6)	8085 (8.6)	
2002	9766 (9.9)	9562 (6.0)		9010 (9.6)	9013 (9.6)	

© 2022 Yunusa I et al. *JAMA Network Open.*

2003	13 563 (13.8)	17 274 (10.8)		13 450 (14.3)	13 437 (14.3)	
2004	13 211 (13.5)	21 265 (13.3)		13 203 (14.0)	13 198 (14.0)	
2005	6767 (6.9)	12 196 (7.6)		6766 (7.2)	6766 (7.2)	
2006	6248 (6.4)	12 080 (7.6)		6247 (6.6)	6239 (6.6)	
2007	4926 (5.0)	9777 (6.1)		4926 (5.2)	4916 (5.2)	
2008	5232 (5.3)	10 949 (6.9)		5232 (5.6)	5233 (5.6)	
2009	5297 (5.4)	11 722 (7.3)		5297 (5.6)	5304 (5.6)	
2010	4528 (4.6)	10 548 (6.6)		4528 (4.8)	4518 (4.8)	
2011	5330 (5.4)	12 727 (8.0)		5330 (5.7)	5327 (5.7)	
2012	4557 (4.6)	11 070 (6.9)		4557 (4.8)	4568 (4.9)	
2013	2595 (2.6)	6289 (3.9)		2595 (2.8)	2598 (2.8)	
2014	1062 (1.1)	2255 (1.4)		1062 (1.1)	1065 (1.1)	
**Healthcare utilization**						
Number of distinct generics, mean (SD)	12.32 (7.36)	12.29 (7.49)	0.003	12.31 (7.38)	12.31 (7.33)	<0.001
Number of physician visits, mean (SD)	4.39 (4.59)	4.38 (4.49)	0.001	4.38 (4.59)	4.39 (4.48)	0.001
Number of hospitalizations, mean, (SD)	0.52 (0.96)	0.57 (1.02)	0.047	0.53 (0.96)	0.52 (0.96)	0.002
Number of hospital days for hospitalizations, mean (SD)	3.43 (9.53)	4.00 (11.14)	0.055	3.48 (9.66)	3.47 (9.63)	0.001
Hospitalization in 30 days before index date	21 131 (21.5)	37 355 (23.4)	0.045	20 468 (21.8)	20 389 (21.7)	0.002
**Comorbidities**						
Combined comorbidity score, mean ±SD	1.04 (1.71)	1.14 (1.85)	0.058	1.05 (1.73)	1.05 (1.72)	<0.001
Alcohol abuse/dependence	1973 (2.0)	3557 (2.2)	0.015	1895 (2.0)	1894 (2.0)	<0.001
Anxiety	20 904 (21.3)	33 580 (21.0)	0.006	19 959 (21.2)	19 976 (21.3)	0.001
Back and neck pain	37 503 (38.2)	59 309 (37.2)	0.021	35 756 (38.0)	35 790 (38.1)	0.001
Bipolar disorder	10 335 (10.5)	17 888 (11.2)	0.022	10 001 (10.6)	10 076 (10.7)	0.003
Back pain- without radiculopathy	35 438 (36.1)	55 734 (34.9)	0.025	33 755 (35.9)	33 764 (35.9)	<0.001
Back pain- with radiculopathy	6798 (6.9)	10 590 (6.6)	0.012	6478 (6.9)	6498 (6.9)	0.001
Bone fracture	34 (0.0)	77 (0.0)	0.007	33.6 (0.0)	34 (0.0)	0.001
Cancer	6087 (6.2)	10 515 (6.6)	0.016	5861 (6.2)	5849 (6.2)	<0.001
COPD/Asthma/Oxygen Use	20 423 (20.8)	33 360 (20.9)	0.002	19 543 (20.8)	19 519 (20.8)	0.001

© 2022 Yunusa I et al. *JAMA Network Open.*

Dementia	1258 (1.3)	2941 (1.8)	0.045	1247 (1.3)	1235 (1.3)	0.001
Dental Pain	7476 (7.6)	11 927 (7.5)	0.005	7132 (7.6)	7179 (7.6)	0.002
Depression	32 789 (33.4)	56 665 (35.5)	0.044	31 672 (33.7)	31 785 (33.8)	0.003
Diabetes	16 108 (16.4)	26 699 (16.7)	0.009	15 446 (16.4)	15 408 (16.4)	0.001
Diabetic neuropathy	2162 (2.2)	3807 (2.4)	0.012	2089 (2.2)	2080 (2.2)	0.001
Epilepsy or convulsions	6344 (6.5)	10 765 (6.7)	0.011	6102 (6.5)	6106 (6.5)	<0.001
Fibromyalgia	7901 (8.0)	12 082 (7.6)	0.018	7488 (8.0)	7531 (8.0)	0.002
Headache	18 872 (19.2)	30 218 (18.9)	0.007	18 026 (19.2)	18 052 (19.2)	0.001
Intentional Self-Harm	303 (0.3)	585 (0.4)	0.01	296 (0.3)	294 (0.3)	<0.001
Liver Disease	8533 (8.7)	15 309 (9.6)	0.031	8259 (8.8)	8249 (8.8)	<0.001
Musculoskeletal Injury	10 688 (10.9)	16 846 (10.6)	0.011	10 185 (10.8)	10 211 (10.9)	0.001
Opioid Dependence/Abuse	3894 (4.0)	6951 (4.4)	0.019	3763 (4.0)	3759 (4.0)	<0.001
Osteoarthritis	10 763 (11.0)	16 732 (10.5)	0.016	10 225 (10.9)	10 192 (10.8)	0.001
Other arthritis, arthropathies and musculoskeletal pain	42 506 (43.3)	69 261 (43.4)	0.002	40 631 (43.2)	40 653 (43.3)	0.001
Other neuropathic pain	15 059 (15.3)	24 048 (15.1)	0.008	14 391 (15.3)	14 423 (15.3)	0.001
Other drug abuse or dependence	7890 (8.0)	14 223 (8.9)	0.031	7637 (8.1)	7639 (8.1)	<0.001
Postherpetic neuralgia	84 (0.1)	118 (0.1)	0.004	77 (0.1)	74 (0.1)	0.001
Previous overdose excl index	393 (0.4)	624 (0.4)	0.001	373 (0.4)	371 (0.4)	<0.001
Psychosis	7215 (7.3)	12 373 (7.8)	0.015	6945 (7.4)	6959 (7.4)	0.001
Renal Dysfunction	5112 (5.2)	10 050 (6.3)	0.047	5001 (5.3)	4974 (5.3)	0.001
Rheumatoid arthritis	1709 (1.7)	2544 (1.6)	0.011	1603 (1.7)	1612 (1.7)	0.001
Tobacco	12 732 (13.0)	23 309 (14.6)	0.047	12 433 (13.2)	12 467 (13.3)	0.001
Urinary calculus	3686 (3.8)	6008 (3.8)	0.001	3540 (3.8)	3555 (3.8)	0.001
**Opioid-related medication use**						
Total oxycodone MME on index date, mean (SD)	454.35 (658.86)	437.06 (629.61)	0.027	449.10 (650.83)	449.17 (650.80)	<0.001
Total Non-oxycodone MME on index date, mean (SD)	41.82 (229.43)	41.84 (231.12)	<0.001	41.86 (230.12)	41.96 (229.76)	<0.001
Total MME 60 days prior to index date, mean (SD)	949.59(2235.30)	908.63(2225.07)	0.018	943.08(2232.82)	942.71 (2238.13)	<0.001
Total MME 180 days prior to index date, mean (SD)	2625.83(6330.39)	2541.38(6388.17)	0.013	2610.45(6329.72)	2606.46(6372.79)	0.001
Codeine 60 pre-index date	8744 (8.9)	12 208 (7.6)	0.046	8077 (8.6)	8073 (8.6)	<0.001

© 2022 Yunusa I et al. *JAMA Network Open.*

Codeine 180 pre-index date	15 804 (16.1)	22 915 (14.4)	0.048	14 734 (15.7)	14 749 (15.7)	0.001
Codeine on index date	503 (0.5)	657 (0.4)	0.015	455 (0.5)	458 (0.5)	<0.001
Hydrocodone 60 pre-index date	38 130 (38.8)	60 010 (37.6)	0.026	36 425 (38.8)	36 484 (38.8)	0.001
Hydrocodone 180 pre-index date	49 232 (50.1)	77 788 (48.7)	0.028	47 057 (50.1)	47 116 (50.1)	0.001
Hydrocodone on index date	2386 (2.4)	3526 (2.2)	0.015	2246 (2.4)	2257 (2.4)	0.001
Hydromorphone 60 pre-index date	1109 (1.1)	2165 (1.4)	0.02	1085 (1.2)	1085 (1.2)	<0.001
Hydromorphone 180 pre-index date	1686 (1.7)	3351 (2.1)	0.028	1654 (1.8)	1652 (1.8)	<0.001
Hydromorphone on index date	317 (0.3)	612 (0.4)	0.01	313 (0.3)	312 (0.3)	<0.001
Levorphanol 60 pre-index date	<11	<11	0.002	<11	<11	<0.001
Levorphanol 180 pre-index date	<11	<11	0.001	<11	<11	<0.001
Levorphanol on index date	<11	<11	0.001	<11	<11	<0.001
Meperidine 60 pre-index date	501 (0.5)	691 (0.4)	0.011	468 (0.5)	474 (0.5)	0.001
Meperidine 180 pre-index	871 (0.9)	1239 (0.8)	0.012	816 (0.9)	820 (0.9)	<0.001
Meperidine on index date	92 (0.1)	113 (0.1)	0.008	84 (0.1)	83 (0.1)	<0.001
Methadone 60 pre-index date	1474 (1.5)	2215 (1.4)	0.01	1400 (1.5)	1399 (1.5)	<0.001
Methadone 180 pre-index date	1920 (2.0)	2890 (1.8)	0.011	1826 (1.9)	1823 (1.9)	<0.001
Methadone on index date	372 (0.4)	599 (0.4)	0.001	355 (0.4)	351 (0.4)	0.001
Morphine 60 pre-index date	2424 (2.5)	3979 (2.5)	0.002	2331 (2.5)	2341 (2.5)	0.001
Morphine 180 pre-index date	3200 (3.3)	5345 (3.3)	0.005	3083 (3.3)	3090 (3.3)	<0.001
Morphine on index date	751 (0.8)	1260 (0.8)	0.003	722 (0.8)	726 (0.8)	<0.001
Naloxone 60 pre-index date	561 (0.6)	1019 (0.6)	0.009	544 (0.6)	549 (0.6)	0.001
Naloxone 180 pre-index date	854 (0.9)	1538 (1.0)	0.01	826 (0.9)	831 (0.9)	0.001
Naloxone on index date	18 (0.0)	50 (0.0)	0.008	17 (0.0)	18 (0.0)	0.001
Oxymorphone 60 pre-index date	46 (0.0)	107 (0.1)	0.008	46 (0.0)	46 (0.0)	<0.001
Oxymorphone 180 pre-index date	61 (0.1)	149 (0.1)	0.011	61 (0.1)	61 (0.1)	<0.001
Oxymorphone on index date	<11	18 (0.0)	0.002	<11	<11	<0.001
Pentazocine 60 pre-index date	333 (0.3)	435 (0.3)	0.012	310 (0.3)	313 (0.3)	<0.001
Pentazocine 180 pre-index	594 (0.6)	809 (0.5)	0.013	559.8 (0.6)	562 (0.6)	<0.001
Pentazocine on index date	11 (0.0)	13 (0.0)	0.003	<11	<11	<0.001

© 2022 Yunusa I et al. *JAMA Network Open.*

Propoxyphene 60 pre-index date	8415 (8.6)	11 488 (7.2)	0.051	7889 (8.4)	7862 (8.4)	0.001
Propoxyphene 180 pre-index date	13 888 (14.1)	19 552 (12.2)	0.056	13 102 (13.9)	13 077 (13.9)	0.001
Propoxyphene on index date	441 (0.4)	624 (0.4)	0.009	415 (0.4)	415 (0.4)	<0.001
Tramadol 60 pre-index date	9666 (9.8)	16 904 (10.6)	0.025	9359 (10.0)	9347 (9.9)	<0.001
Tramadol 180 pre-index date	15 960 (16.3)	27 216 (17.0)	0.021	15 411 (16.4)	15 428 (16.4)	0.001
Tramadol on index date	671 (0.7)	1262 (0.8)	0.013	651 (0.7)	651 (0.7)	<0.001
Fentanyl 60 pre-index date	2711 (2.8)	4539 (2.8)	0.005	2617 (2.8)	2614 (2.8)	<0.001
Fentanyl 180 pre-index date	3622 (3.7)	6238 (3.9)	0.011	3508 (3.7)	3506 (3.7)	<0.001
Fentanyl on index date	1074 (1.1)	2018 (1.3)	0.016	1056 (1.1)	1063 (1.1)	0.001
Tapentadol 60 pre-index date	18 (0.0)	73 (0.0)	0.015	18 (0.0)	18 (0.0)	<0.001
Tapentadol 180 pre-index date	31 (0.0)	125 (0.1)	0.02	31 (0.0)	31 (0.0)	<0.001
Tapentadol on index date	<11	<11	0.006	<11	<11	<0.001
Dihydrocodeine 60 pre-index date	227 (0.2)	331 (0.2)	0.005	217 (0.2)	216 (0.2)	<0.001
Dihydrocodeine 180 pre-index date	503 (0.5)	653 (0.4)	0.015	469 (0.5)	469 (0.5)	<0.001
Dihydrocodeine on index date	14 (0.0)	<11	0.01	<11	<11	0.001
Butorphanol 60 pre-index date	274 (0.3)	322 (0.2)	0.016	244 (0.3)	247 (0.3)	0.001
Butorphanol 180 pre-index date	377 (0.4)	459 (0.3)	0.017	340 (0.4)	344 (0.4)	0.001
Butorphanol on index date	33 (0.0)	32 (0.0)	0.008	25 (0.0)	26 (0.0)	<0.001
Buprenorphine 60 pre-index date	369 (0.4)	822 (0.5)	0.021	368 (0.4)	368 (0.4)	<0.001
Buprenorphine 180 pre-index date	465 (0.5)	1065 (0.7)	0.026	464 (0.5)	464 (0.5)	<0.001
Buprenorphine on index date	13 (0.0)	51 (0.0)	0.012	13 (0.0)	13 (0.0)	<0.001
**Other Prior medications**						
SSRI 180 pre-index	94 229 (96.0)	151 117 (94.7)	0.062	90 079 (95.8)	90 069 (95.8)	<0.001
Other antidepressants 180 plus index	36 104 (36.8)	57 558 (36.1)	0.015	34 422 (36.6)	34 553 (36.8)	0.003
Benzodiazepines 180 plus index	45 082 (45.9)	71 329 (44.7)	0.025	42 955 (45.7)	43 012 (45.8)	0.001
Muscle Relaxants 180 plus index	29 903 (30.5)	47 965 (30.0)	0.009	28 650 (30.5)	28 669 (30.5)	0.001
Other Sedatives-Hypnotics 180 plus index	15 709 (16.0)	27 458 (17.2)	0.032	15 225 (16.2)	15 230 (16.2)	<0.001
NSAID 180 plus index	50 261 (51.2)	79 121 (49.6)	0.032	47 846 (50.9)	47 838 (50.9)	<0.001
Lithium 180 plus index	1871 (1.9)	2763 (1.7)	0.013	1752 (1.9)	1765 (1.9)	0.001

© 2022 Yunusa I et al. *JAMA Network Open.*

Atypical antipsychotics 180 plus index	21 601 (22.0)	37 271 (23.3)	0.032	20 988 (22.3)	21 097 (22.4)	0.003
Typical antipsychotics 180 plus index	2537 (2.6)	3961 (2.5)	0.007	2376 (2.5)	2381 (2.5)	<0.001
Barbiturates 180 plus index	885 (0.9)	1205 (0.8)	0.016	816 (0.9)	817 (0.9)	<0.001
Agents for dementia 180 plus index	410 (0.4)	931 (0.6)	0.023	406 (0.4)	402 (0.4)	0.001
Anticonvulsants 180 plus index	16 741 (17.0)	28 980 (18.2)	0.029	16 214 (17.2)	16 302 (17.3)	0.003
Gabapentinoids 180 plus index	16 184 (16.5)	28 012 (17.5)	0.028	15 630 (16.6)	15 672 (16.7)	0.001
Triptans 180 plus index	5279 (5.4)	8216 (5.1)	0.01	5030 (5.4)	5044 (5.4)	0.001
CYP 3A4 inhibitors overlapping index	10 503 (10.7)	17 557 (11.0)	0.01	10 065 (10.7)	10 015 (10.7)	0.002
CYP 3A4 inducers overlapping index	7841 (8.0)	13 400 (8.4)	0.015	7590 (8.1)	7606 (8.1)	0.001
CYP 2D6 inhibitors overlapping index	2196 (2.2)	4159 (2.6)	0.024	2137 (2.3)	2117 (2.3)	0.001
CYP 2D6 inducers overlapping index	4282 (4.4)	7288 (4.6)	0.01	4106 (4.3)	4068 (4.3)	0.002

Abbreviations: SSRI, selective serotonin reuptake inhibitors; SD, standard deviation; COPD, chronic obstructive pulmonary disease; MME, morphine milligram equivalent; Std. diff., standardized difference; NSAID, Nonsteroidal anti-inflammatory drugs; CYP, cytochrome P450

[a]Counts less than 11 were suppressed per Centers for Medicare & Medicaid Services policy.

[b]Standardized differences greater than 0.1 indicate lack of balance.

© 2022 Yunusa I et al. *JAMA Network Open.*

eTable 6. Patient Baseline Characteristics Before and After Propensity Score Weighting for Optum Database

Patient characteristic	SSRI Exposure groups					
	Unweighted			Weighted		
	**CPY2D6-inhibiting SSRI**	**Other SSRI**	**[a]Std. diff.**	**CPY2D6-inhibiting SSRI**	**Other SSRI**	**[a]Std. diff.**
**Number of patients**	183 000	443 178		182 960	182 956	
**Demographics**						
Age, mean (SD), y	53.99 (16.18)	54.02 (15.09)	0.002	54.02 (15.09)	54.00 (15.19)	0.002
Age category, y			0.122			0.003
18-24	5787 (3.2)	15 804 (3.6)		5786 (3.2)	5823 (3.2)	
25-34	15 500 (8.5)	44 976 (10.1)		15 499 (8.5)	15 462 (8.5)	
35-44	28 515 (15.6)	70 729 (16.0)		28 511 (15.6)	28 532 (15.6)	
45-54	40 160 (21.9)	89 046 (20.1)		40 149 (21.9)	40 269 (22.0)	
55-64	42 276 (23.1)	92 007 (20.8)		42 259 (23.1)	42 305 (23.1)	
65-74	34 979 (19.1)	80 943 (18.3)		34 972 (19.1)	34 868 (19.1)	
75 and above	15 783 (8.6)	49 673 (11.2)		15 783 (8.6)	15 694 (8.6)	
Sex			0.047			0.001
Female	133 135 (72.8)	313 008 (70.6)		133 100 (72.7)	133 040 (72.7)	
Region			0.06			0.001
Midwest	41 498 (22.7)	102 766 (23.2)		41 493 (22.7)	41 468 (22.7)	
Northeast	19 190 (10.5)	50 465 (11.4)		19 188 (10.5)	19 192 (10.5)	
South	76 336 (41.7)	189 322 (42.7)		76 324 (41.7)	76 409 (41.8)	
West	45 849 (25.1)	100 329 (22.6)		45 827 (25.0)	45 760 (25.0)	
Unknown	127 (0.1)	296 (0.1)		127 (0.1)	125 (0.1)	
Calendar year of index date			0.197			0.001
2004	5776 (3.2)	7938 (1.8)		5757 (3.1)	5756 (3.1)	

© 2022 Yunusa I et al. *JAMA Network Open.*

2005	10 476 (5.7)	16 325 (3.7)		10 465 (5.7)	10 476 (5.7)	
2006	13 106 (7.2)	22 986 (5.2)		13 100 (7.2)	13 077 (7.1)	
2007	12 079 (6.6)	23 637 (5.3)		12 077 (6.6)	12 062 (6.6)	
2008	11 553 (6.3)	24 927 (5.6)		11 551 (6.3)	11 546 (6.3)	
2009	11 049 (6.0)	25 314 (5.7)		11 048 (6.0)	11 046 (6.0)	
2010	11 202 (6.1)	26 602 (6.0)		11 202 (6.1)	11 192 (6.1)	
2011	11 352 (6.2)	28 048 (6.3)		11 351 (6.2)	11 356 (6.2)	
2012	11 155 (6.1)	28 308 (6.4)		11 155 (6.1)	11 167 (6.1)	
2013	11 279 (6.2)	29 414 (6.6)		11 279 (6.2)	11 289 (6.2)	
2014	10 218 (5.6)	27 204 (6.1)		10 218 (5.6)	10 208 (5.6)	
2015	11 120 (6.1)	30 234 (6.8)		11 120 (6.1)	11 122 (6.1)	
2016	12 156 (6.6)	33 539 (7.6)		12 156 (6.6)	12 158 (6.6)	
2017	12 626 (6.9)	35 681 (8.1)		12 626 (6.9)	12 636 (6.9)	
2018	11 521 (6.3)	33 884 (7.6)		11 521 (6.3)	11 527 (6.3)	
2019	11 774 (6.4)	34 864 (7.9)		11 774 (6.4)	11 776 (6.4)	
2020	4558 (2.5)	14 273 (3.2)		4558 (2.5)	4555 (2.5)	
**Healthcare utilization**						
Number of distinct generics, mean (SD)	9.09 (5.46)	9.03 (5.54)	0.012	9.09 (5.46)	9.11 (5.49)	0.003
Number of physician visits, mean (SD)	5.56 (4.25)	5.64 (4.35)	0.02	5.56 (4.25)	5.57 (4.26)	0.003
Number of hospitalizations, mean, (SD)	0.37 (0.95)	0.43 (1.14)	0.063	0.37 (0.95)	0.37 (0.93)	<0.001
Number of hospital days for hospitalizations, mean (SD)	1.92 (6.41)	2.38 (8.06)	0.063	1.92 (6.41)	1.93 (6.45)	0.001
Hospitalization in 30 days before index date	35 685 (19.5)	92 321 (20.8)	0.033	35 681 (19.5)	35 608 (19.5)	0.001
**Comorbidities**						
Combined comorbidity score, mean (SD)	1.16 (2.20)	1.37 (2.44)	0.092	1.16 (2.20)	1.16 (2.20)	0.001
Alcohol abuse/dependence	3059 (1.7)	7757 (1.8)	0.006	3058 (1.7)	3068 (1.7)	<0.001
Anxiety	54 759 (29.9)	145 753 (32.9)	0.064	54 754 (29.9)	54 833 (30.0)	0.001
Back and neck pain	69 454 (38.0)	167 014 (37.7)	0.006	69 436 (38.0)	69 570 (38.0)	0.002
Bipolar disorder	8173 (4.5)	15 656 (3.5)	0.048	8158 (4.5)	8278 (4.5)	0.003
Back pain- without radiculopathy	62 002 (33.9)	147 998 (33.4)	0.01	61 985 (33.9)	62 111 (33.9)	0.001

© 2022 Yunusa I et al. *JAMA Network Open.*

Back pain- with radiculopathy	20 772 (11.4)	48 239 (10.9)	0.015	20 765 (11.3)	20 833 (11.4)	0.001
Bone fracture	8667 (4.7)	25 455 (5.7)	0.045	8667 (4.7)	8670 (4.7)	<0.001
Cancer	17 264 (9.4)	46 159 (10.4)	0.033	17 262 (9.4)	17 263 (9.4)	<0.001
COPD/Asthma/Oxygen Use	33 535 (18.3)	81 730 (18.4)	0.003	33 527 (18.3)	33 564 (18.3)	0.001
Dementia	6070 (3.3)	23 026 (5.2)	0.093	6069 (3.3)	6086 (3.3)	0.001
Dental Pain	1733 (0.9)	3884 (0.9)	0.007	1732 (0.9)	1736 (0.9)	<0.001
Depression	65 028 (35.5)	164 975 (37.2)	0.035	65 020 (35.5)	65 261 (35.7)	0.003
Diabetes	35 156 (19.2)	86 108 (19.4)	0.006	35 148 (19.2)	35 140 (19.2)	<0.001
Diabetic neuropathy	6937 (3.8)	18 266 (4.1)	0.017	6935 (3.8)	6957 (3.8)	0.001
Epilepsy or convulsions	4201 (2.3)	10 858 (2.5)	0.01	4199 (2.3)	4229 (2.3)	0.001
Fibromyalgia	14 554 (8.0)	31 337 (7.1)	0.033	14 546 (8.0)	14 621 (8.0)	0.002
Headache	27 363 (15.0)	65 205 (14.7)	0.007	27 354 (15.0)	27 471 (15.0)	0.002
Intentional Self-Harm	883 (0.5)	2147 (0.5)	<0.001	882 (0.5)	887 (0.5)	<0.001
Liver Disease	12 503 (6.8)	32 416 (7.3)	0.019	12 502 (6.8)	12 535 (6.9)	0.001
Musculoskeletal Injury	23 420 (12.8)	55 503 (12.5)	0.008	23 414 (12.8)	23 465 (12.8)	0.001
Opioid Dependence/Abuse	1582 (0.9)	3285 (0.7)	0.014	1580 (0.9)	1595 (0.9)	0.001
Osteoarthritis	42 060 (23.0)	101 935 (23.0)	<0.001	42 053 (23.0)	42 075 (23.0)	<0.001
Other arthritis, arthropathies and musculoskeletal pain	93 506 (51.1)	229 512 (51.8)	0.014	93 489 (51.1)	93 566 (51.1)	0.001
Other neuropathic pain	38 093 (20.8)	91 361 (20.6)	0.005	38 083 (20.8)	38 166 (20.9)	0.001
Other drug abuse or dependence	2949 (1.6)	6316 (1.4)	0.015	2946 (1.6)	2976 (1.6)	0.001
Postherpetic neuralgia	311 (0.2)	925 (0.2)	0.009	311 (0.2)	309 (0.2)	<0.001
Previous overdose excl index	245 (0.1)	559 (0.1)	0.002	245 (0.1)	244 (0.1)	<0.001
Psychosis	3530 (1.9)	9427 (2.1)	0.014	3529 (1.9)	3558 (1.9)	0.001
Renal Dysfunction	16 249 (8.9)	44 601 (10.1)	0.04	16 247 (8.9)	16 252 (8.9)	<0.001
Rheumatoid arthritis	4796 (2.6)	11 171 (2.5)	0.006	4794 (2.6)	4798 (2.6)	<0.001
Tobacco	32 934 (18.0)	81 382 (18.4)	0.01	32 928 (18.0)	32 988 (18.0)	0.001
Urinary calculus	10 359 (5.7)	26 051 (5.9)	0.009	10 358 (5.7)	10 375 (5.7)	<0.001
**Opioid-related medication use**						
Total oxycodone MME on index date, mean (SD)	384.53(444.53)	372.10 426.28)	0.029	384.48(444.45)	384.65(444.72)	0.001

© 2022 Yunusa I et al. *JAMA Network Open.*

Total Non-oxycodone MME on index date, mean (SD)	22.63(127.79)	21.53(123.66)	0.001	22.62(127.75)	22.67(127.77)	<0.001
Total MME 60 days prior to index date, mean (SD)	471.22(1343.71)	413.78(1242.93)	0.005	470.84(1342.83)	471.24(1348.23)	<0.001
Total MME 180 days prior to index date, mean (SD)	1344.22(3962.19)	1175.41(3649.76)	0.004	1343.10(3959.61)	1345.29(3977.90)	0.001
Codeine 60 pre-index date	6734 (3.7)	15 245 (3.4)	0.013	6730 (3.7)	6741 (3.7)	<0.001
Codeine 180 pre-index date	13 208 (7.2)	30 456 (6.9)	0.013	13 202 (7.2)	13 219 (7.2)	<0.001
Codeine on index date	435 (0.2)	986 (0.2)	0.003	434 (0.2)	433 (0.2)	<0.001
Hydrocodone 60 pre-index date	49 556 (27.1)	108 371 (24.5)	0.06	49 534 (27.1)	49 556 (27.1)	<0.001
Hydrocodone 180 pre-index date	66 224 (36.2)	147 496 (33.3)	0.061	66 197 (36.2)	66 215 (36.2)	<0.001
Hydrocodone on index date	3701 (2.0)	8276 (1.9)	0.011	3700 (2.0)	3698 (2.0)	<0.001
Hydromorphone 60 pre-index date	1451 (0.8)	3462 (0.8)	0.001	1449 (0.8)	1453 (0.8)	<0.001
Hydromorphone 180 pre-index date	2188 (1.2)	5136 (1.2)	0.003	2186 (1.2)	2191 (1.2)	<0.001
Hydromorphone on index date	429 (0.2)	980 (0.2)	0.003	429 (0.2)	431 (0.2)	<0.001
Levorphanol 60 pre-index date	7 (0.0)	16 (0.0)	<0.001	6 (0.0)	7 (0.0)	<0.001
Levorphanol 180 pre-index date	11 (0.0)	23 (0.0)	0.001	10 (0.0)	11 (0.0)	<0.001
Levorphanol on index date	1 (0.0)	2 (0.0)	<0.001	1 (0.0)	1 (0.0)	<0.001
Meperidine 60 pre-index date	469 (0.3)	930 (0.2)	0.01	468 (0.3)	471 (0.3)	<0.001
Meperidine 180 pre-index	814 (0.4)	1580 (0.4)	0.014	813 (0.4)	817 (0.4)	<0.001
Meperidine on index date	115 (0.1)	279 (0.1)	<0.001	114 (0.1)	116 (0.1)	<0.001
Methadone 60 pre-index date	1098 (0.6)	1953 (0.4)	0.022	1095 (0.6)	1105 (0.6)	0.001
Methadone 180 pre-index date	1361 (0.7)	2463 (0.6)	0.023	1358 (0.7)	1372 (0.8)	0.001
Methadone on index date	265 (0.1)	500 (0.1)	0.009	264 (0.1)	267 (0.1)	<0.001
Morphine 60 pre-index date	2696 (1.5)	5794 (1.3)	0.014	2692 (1.5)	2691 (1.5)	<0.001
Morphine 180 pre-index date	3476 (1.9)	7494 (1.7)	0.016	3471 (1.9)	3471 (1.9)	<0.001
Morphine on index date	1147 (0.6)	2590 (0.6)	0.005	1146 (0.6)	1149 (0.6)	<0.001
Naloxone 60 pre-index date	634 (0.3)	1287 (0.3)	0.01	633 (0.3)	636 (0.3)	<0.001
Naloxone 180 pre-index date	947 (0.5)	2006 (0.5)	0.009	946 (0.5)	954 (0.5)	0.001
Naloxone on index date	161 (0.1)	408 (0.1)	0.001	160 (0.1)	159 (0.1)	<0.001
Oxymorphone 60 pre-index date	294 (0.2)	813 (0.2)	0.005	294 (0.2)	294 (0.2)	<0.001
Oxymorphone 180 pre-index date	411 (0.2)	1075 (0.2)	0.004	411 (0.2)	412 (0.2)	<0.001

© 2022 Yunusa I et al. *JAMA Network Open.*

Oxymorphone on index date	58 (0.0)	161 (0.0)	0.003	58 (0.0)	59(0.0)	0.001
Pentazocine 60 pre-index date	125 (0.1)	214 (0.0)	0.008	124 (0.1)	124 (0.1)	<0.001
Pentazocine 180 pre-index	200 (0.1)	356 (0.1)	0.009	199 (0.1)	201 (0.1)	<0.001
Pentazocine on index date	5 (0.0)	18 (0.0)	0.002	5 (0.0)	5 (0.0)	<0.001
Propoxyphene 60 pre-index date	4402 (2.4)	7593 (1.7)	0.049	4393 (2.4)	4392 (2.4)	<0.001
Propoxyphene 180 pre-index date	7178 (3.9)	12 823 (2.9)	0.057	7167 (3.9)	7163 (3.9)	<0.001
Propoxyphene on index date	383 (0.2)	741 (0.2)	0.01	382 (0.2)	381 (0.2)	<0.001
Tramadol 60 pre-index date	12 853 (7.0)	34 089 (7.7)	0.026	12 852 (7.0)	12 882 (7.0)	0.001
Tramadol 180 pre-index date	20 258 (11.1)	52 144 (11.8)	0.022	20 255 (11.1)	20 312 (11.1)	0.001
Tramadol on index date	1932 (1.1)	5102 (1.2)	0.009	1932.0 (1.1)	1939 (1.1)	<0.001
Fentanyl 60 pre-index date	2166 (1.2)	5014 (1.1)	0.005	2164 (1.2)	2167 (1.2)	<0.001
Fentanyl 180 pre-index date	2718 (1.5)	6343 (1.4)	0.005	2715 (1.5)	2726 (1.5)	<0.001
Fentanyl on index date	809 (0.4)	1975 (0.4)	0.001	808 (0.4)	805 (0.4)	<0.001
Tapentadol 60 pre-index date	312 (0.2)	767 (0.2)	0.001	312 (0.2)	313 (0.2)	<0.001
Tapentadol 180 pre-index date	470 (0.3)	1180 (0.3)	0.002	470 (0.3)	474 (0.3)	<0.001
Tapentadol on index date	29 (0.0)	99 (0.0)	0.005	29 (0.0)	29 (0.0)	<0.001
Dihydrocodeine 60 pre-index date	55 (0.0)	108 (0.0)	0.003	54 (0.0)	54 (0.0)	<0.001
Dihydrocodeine 180 pre-index date	117 (0.1)	234 (0.1)	0.005	116 (0.1)	117 (0.1)	<0.001
Dihydrocodeine on index date	5 (0.0)	3 (0.0)	0.005	3 (0.0)	3 (0.0)	<0.001
Butorphanol 60 pre-index date	166 (0.1)	291 (0.1)	0.009	165 (0.1)	167 (0.1)	<0.001
Butorphanol 180 pre-index date	210 (0.1)	389 (0.1)	0.008	209 (0.1)	212 (0.1)	<0.001
Butorphanol on index date	10 (0.0)	26 (0.0)	0.001	10 (0.0)	10 (0.0)	<0.001
Buprenorphine 60 pre-index date	740 (0.4)	1513 (0.3)	0.01	739 (0.4)	740 (0.4)	<0.001
Buprenorphine 180 pre-index date	1005 (0.5)	2122 (0.5)	0.01	1004 (0.5)	1008 (0.6)	<0.001
Buprenorphine on index date	49 (0.0)	96 (0.0)	0.003	48 (0.0)	48 (0.0)	<0.001
**Other Prior medications**						
SSRI 180 pre-index	180 162 (98.4)	432 709 (97.6)	0.059	180 122 (98.4)	180 124 (98.5)	<0.001
Other antidepressants 180 plus index	47 117 (25.7)	108 768 (24.5)	0.028	47 093 (25.7)	47 332 (25.9)	0.003
Benzodiazepines 180 plus index	61 089 (33.4)	144 731 (32.7)	0.015	61 064 (33.4)	61 244 (33.5)	0.002

© 2022 Yunusa I et al. *JAMA Network Open.*

Muscle Relaxants 180 plus index	38 433 (21.0)	87 071 (19.6)	0.034	38 417 (21.0)	38 500 (21.0)	0.001
Other Sedatives-Hypnotics 180 plus index	22 352 (12.2)	55 564 (12.5)	0.01	22 347 (12.2)	22 469 (12.3)	0.002
NSAID 180 plus index	63 536 (34.7)	148 891 (33.6)	0.024	63 514 (34.7)	63 569 (34.7)	0.001
Lithium 180 plus index	1374 (0.8)	2338 (0.5)	0.028	1365 (0.7)	1387 (0.8)	0.001
Atypical antipsychotics 180 plus index	13 300 (7.3)	27 719 (6.3)	0.04	13 279 (7.3)	13 397 (7.3)	0.002
Typical antipsychotics 180 plus index	897 (0.5)	2140 (0.5)	0.001	896 (0.5)	901 (0.5)	<0.001
Barbiturates 180 plus index	680 (0.4)	1213 (0.3)	0.017	677 (0.4)	677 (0.4)	<0.001
Agents for dementia 180 plus index	2688 (1.5)	10 895 (2.5)	0.071	2688 (1.5)	2688 (1.5)	<0.001
Anticonvulsants 180 plus index	17 344 (9.5)	39 371 (8.9)	0.021	17 328 (9.5)	17 514 (9.6)	0.003
Gabapentinoids 180 plus index	27 526 (15.0)	66 600 (15.0)	<0.001	27 517 (15.0)	27 626 (15.1)	0.002
Triptans 180 plus index	7868 (4.3)	16 923 (3.8)	0.024	7863 (4.3)	7933 (4.3)	0.002
CYP 3A4 inhibitors overlapping index	15 250 (8.3)	37 518 (8.5)	0.005	15 244 (8.3)	15 244 (8.3)	<0.001
CYP 3A4 inducers overlapping index	7706 (4.2)	17 078 (3.9)	0.018	7699 (4.2)	7802 (4.3)	0.003
CYP 2D6 inhibitors overlapping index	1869 (1.0)	5055 (1.1)	0.012	1868 (1.0)	1872 (1.0)	<0.001
CYP 2D6 inducers overlapping index	2034 (1.1)	4572 (1.0)	0.008	2031 (1.1)	2037 (1.1)	<0.001

Abbreviations: SSRI, selective serotonin reuptake inhibitors; SD, standard deviation; COPD, chronic obstructive pulmonary disease; MME, morphine milligram equivalent; Std. diff., standardized difference; NSAID, Nonsteroidal anti-inflammatory drugs; CYP, cytochrome P450
[a]Standardized differences greater than 0.1 indicate lack of balance.

© 2022 Yunusa I et al. *JAMA Network Open.*

eTable 7. Follow-up Duration			
**Analysis**	**Exposure**	**Mean (Days)**	**SD (Days)**
As Treated	Other SSRI (crude)	23.20	24.37
	CPY2D6-inhibiting SSRI (crude)	23.64	25.57
	Other SSRI (Weighted)	23.52	25.44
	CPY2D6-inhibiting SSRI (Weighted)	23.61	25.45
ITT	Other SSRI (crude)	57.87	8.88
	CPY2D6-inhibiting SSRI (crude)	57.98	8.67
	Other SSRI (Weighted)	57.96	8.70
	CPY2D6-inhibiting SSRI (Weighted)	57.98	8.67

Abbreviations: SSRI, selective serotonin reuptake inhibitors; SD, standard deviation; COPD, chronic obstructive pulmonary disease; MME, morphine milligram equivalent; Std. diff., standardized difference; NSAID, Non-steroidal anti-inflammatory drugs; CYP, cytochrome P450

© 2022 Yunusa I et al. *JAMA Network Open.*

eTable 8. Reasons for Censoring by Treatment Group Based on Primary Analysis[a]

**MarketScan**			
	**Overall (N=1 153 484)**	**CPY2D6-inhibiting SSRI (N=337 586)**	**Other SSRI (N=815 898)**
**Outcome/reason for censoring**			
Outcome, n(%)	386 (0.03)	137 (0.04)	249 (0.03)
SSRI switch , n(%)	2749 (0.24)	1431 (0.42)	1318 (0.16)
SSRI discontinuation, n(%)	255 399 (22.15)	75 854 (22.48)	179 545 (22.01)
Oxycodone discontinuation, n(%)	874 567 (75.84)	254 571 (75.44)	619 996 (76.01)
Death, n(%)	830 (0.07)	244 (0.07)	586 (0.07)
Enrollment end, n(%)	28 148 (2.44)	7934 (2.35)	20 214 (2.48)
1-year follow-up, n(%)	1110 (0.10)	341 (0.10)	769 (0.09)
End of data, n(%)	3682 (0.32)	968 (0.29)	2714 (0.33)
**MAX**			
	**Overall (N=257 828)**	**CPY2D6-inhibiting SSRI (N=98 192)**	**Other SSRI (N=159 636)**
**Outcome/reason for censoring**			
Outcome, n(%)	326 (0.13)	138 (0.14)	188 (0.12)
SSRI switch, n(%)	1716 (0.67)	874 (0.89)	842 (0.53)
SSRI discontinuation, n(%)	82 042 (31.86)	31 507 (32.13)	50 535 (31.69)
Oxycodone discontinuation, n(%)	166 479 (64.65)	63 053 (64.30)	103 426 (64.87)
Death, n(%)	1002 (0.39)	335 (0.34)	667 (0.42)
Enrollment end, n(%)	8501 (3.30)	3114 (3.18)	5387 (3.38)
1-year follow-up, n(%)	557 (0.22)	219 (0.22)	338 (0.21)
End of data, n(%)	257 (0.10)	89 (0.09)	168 (0.11)
**Optum**			
	**Overall (N=626 178)**	**CPY2D6-inhibiting SSRI (N=183 000)**	**Other SSRI (N=443 178)**
**Outcome/reason for censoring**			
Outcome, n (%)	323 (0.05)	106 (0.06)	217 (0.05)

© 2022 Yunusa I et al. *JAMA Network Open.*

SSRI switch, n (%)	1510 (0.24)	755 (0.41)	755 (0.17)
SSRI discontinuation, n (%)	145 066 (23.18)	43 220 (23.63)	101 846 (22.99)
Oxycodone discontinuation, n (%)	466 684 (74.57)	135 555 (74.12)	331 129 (74.75)
Death, n (%)	1442 (0.23)	312 (0.17)	1130 (0.26)
Enrollment end, n (%)	16 762 (2.68)	4625 (2.53)	12 137 (2.74)
1-year follow-up, n (%)	964 (0.15)	293 (0.16)	671 (0.15)
End of data, n (%)	3116 (0.50)	772 (0.42)	2344 (0.53)
**Combined dataset**			
	**Overall (N=2 037 490)**	**CPY2D6-inhibiting SSRI (N=618 778)**	**Other SSRI (N=1 418 712)**
**Outcome/reason for censoring**			
Outcome, n (%)	1035 (0.05)	381 (0.06)	654 (0.05)
SSRI switch, n (%)	5975 (3.37)	3060 (3.35)	2915 (3.40)
SSRI discontinuation, n (%)	482 507 (27.23)	150 581 (27.83)	331 926 (26.97)
Oxycodone discontinuation, n (%)	1 507 730 (77.08)	453 179 (76.28)	1 054 551 (77.42)
Death, n (%)	3274 (2.92)	891 (2.55)	2383 (3.09)
Enrollment end, n (%)	53 411 (2.62)	15 673 (2.53)	37 738 (2.66)
1-year follow-up, n (%)	2631 (0.13)	853 (0.14)	1778 (0.13)
End of data, n (%)	7055 (0.35)	1829 (0.30)	5226 (0.37)

Abbreviation: SSRI, selective serotonin reuptake inhibitors

[a]Reasons for censoring are not mutually exclusive if more than one occurred on the same date

© 2022 Yunusa I et al. *JAMA Network Open.*

eTable 9. Results for Each Database[a]										
	Exposure	N	Number of events	Follow-up			Incidence Rate *per 1000 PY*	Hazard Ratio		
				Total (Years)	Mean (Days)	SD (Days)		HR	HR LCL	HR UCL
MAX	Other SSRI (crude)	159 636	188	11 024.67	25.22	31.21	17.05	1[Reference]	1[Reference]	1[Reference]
	CPY2D6-inhibiting SSRI (crude)	98 192	138	6850.31	25.48	31.70	20.15	1.17	0.94	1.46
	Other SSRI (weighted)	93 978	109	6545.10	25.44	31.78	16.65	1[Reference]	1[Reference]	1[Reference]
	CPY2D6-inhibiting SSRI (weighted)	93 998	133	6521.01	25.34	31.38	20.40	1.23	0.98	1.54
Optum	Other SSRI (crude)	443 178	217	28 689.18	23.64	25.62	7.56	1[Reference]	1[Reference]	1[Reference]
	Inhibiting SSRI (crude)	183 000	106	12 009.09	23.97	26.47	8.83	1.15	0.91	1.45
	Non-inhibiting SSRI (weighted)	182 956	90	11 936.90	23.83	26.26	7.54	1[Reference]	1[Reference]	1[Reference]
	CPY2D6-inhibiting SSRI (weighted)	182 960	106	12 006.23	23.97	26.47	8.83	1.16	0.91	1.47
MarketScan	Other SSRI (crude)	1 418 712	654	90 125.22	23.20	24.37	7.26	1[Reference]	1[Reference]	1[Reference]
	CPY2D6-inhibiting SSRI (crude)	618 778	381	40 052.75	23.64	25.57	9.51	1.20	1.06	1.36
	Other SSRI (weighted)	614 336	303	39 567.26	23.52	25.44	7.66	1[Reference]	1[Reference]	1[Reference]
	CPY2D6-inhibiting SSRI (weighted)	614 385	376	39 706.85	23.61	25.45	9.47	1.23	1.08	1.40

Abbreviations: SSRI, selective serotonin reuptake inhibitors; SD, standard deviation; HR, hazard ratio; LCL, lower confidence limit; UCL, upper confidence limit; PY, person-years

[a]Results are based on Primary (As Treated) Analysis

© 2022 Yunusa I et al. *JAMA Network Open.*
